# Energy balance-related factors and risk of colorectal cancer based on *KRAS*, *PIK3CA*, and *BRAF* mutations and MMR status

**DOI:** 10.1007/s00432-022-04019-9

**Published:** 2022-05-11

**Authors:** Josien C. A. Jenniskens, Kelly Offermans, Colinda C. J. M. Simons, Iryna Samarska, Gregorio E. Fazzi, Jaleesa R. M. van der Meer, Kim M. Smits, Leo J. Schouten, Matty P. Weijenberg, Heike I. Grabsch, Piet A. van den Brandt

**Affiliations:** 1 Department of Epidemiology, GROW School for Oncology and Reproduction, Maastricht University Medical Center+, P.O. BOX 616, 6200 MD Maastricht, The Netherlands; 2 Department of Pathology, GROW School for Oncology and Reproduction, Maastricht University Medical Center+, P.O. BOX 5800, 6202 AZ Maastricht, The Netherlands; 3 Pathology and Data Analytics, Leeds Institute of Medical Research at St James’s, University of Leeds, Leeds, UK; 4 Department of Epidemiology, Care and Public Health Research Institute (CAPHRI), Maastricht University Medical Center+, Maastricht, The Netherlands

**Keywords:** Prospective cohort study, Energy balance, Colorectal cancer, Mutations, Mismatch repair/microsatellite instability, Etiological heterogeneity

## Abstract

**Introduction:**

*KRAS* mutations (*KRAS*_mut_), *PIK3CA*_mut_, *BRAF*_mut_, and mismatch repair deficiency (dMMR) have been associated with the Warburg-effect. We previously observed differential associations between energy balance-related factors (BMI, clothing-size, physical activity) and colorectal cancer (CRC) subtypes based on the Warburg-effect. We now investigated whether associations between energy balance-related factors and risk of CRC differ between subgroups based on mutation and MMR status.

**Methods:**

Information on molecular features was available for 2349 incident CRC cases within the Netherlands Cohort Study (NLCS), with complete covariate data available for 1934 cases and 3911 subcohort members. Multivariable-adjusted Cox-regression was used to estimate associations of energy balance-related factors with risk of CRC based on individual molecular features (*KRAS*_mut_*; PIK3CA*_mut_; *BRAF*_mut_; dMMR) and combinations thereof (all-wild-type + MMR-proficient (pMMR); any-mutation/dMMR).

**Results:**

In men, BMI and clothing-size were positively associated with risk of colon, but not rectal cancer, regardless of molecular features subgroups; the strongest associations were observed for *PIK3CA*_mut_ colon cancer. In women, however, BMI and clothing-size were only associated with risk of *KRAS*_mut_ colon cancer (*p*-heterogeneity_*KRAS*mut versus all-wild-type+pMMR_ = 0.008). Inverse associations of non-occupational physical activity with risk of colon cancer were strongest for any-mutation/dMMR tumors in men and women, and specifically for *PIK3CA*_mut_ tumors in women. Occupational physical activity was inversely associated with both combination subgroups of colon cancer in men.

**Conclusion:**

In men, associations did not vary according to molecular features. In women, a role of *KRAS* mutations in the etiological pathway between adiposity and colon cancer is suggested, and of *PIK3CA* mutations between physical activity and colon cancer.

**Supplementary Information:**

The online version contains supplementary material available at 10.1007/s00432-022-04019-9.

## Introduction

Colorectal cancer (CRC) risk was shown to be affected by energy balance-related factors (Moghaddam et al. 2007; Robsahm et al. 2013; Wolin et al. 2009; Samad et al. 2005). Adiposity measures, such as body mass index (BMI) and waist circumference, have been associated with an increased risk of CRC (Moghaddam et al. 2007; Robsahm et al. 2013), whereas physical activity has been associated with a decreased risk of CRC (Robsahm et al. 2013; Wolin et al. 2009; Samad et al. 2005). One of the proposed mechanisms underlying these associations is activation of the so-called Warburg-effect through upregulated PI3K/Akt-signaling (Huang and Chen 2009; Levine and Puzio-Kuter 2010; Feron 2009; Schwartz et al. 2017; Hanahan and Weinberg 2011). We have previously observed differential associations between energy balance-related factors (i.e. BMI; clothing-size, as a proxy for waist circumference; physical activity) and CRC subtypes expressing different levels of proteins involved in the Warburg-effect (Jenniskens et al. 2021a).

The Warburg-effect is a metabolic phenotype first discovered in the 1920s by Otto Warburg and colleagues (Warburg 1925). This phenotype is characterized by increased aerobic glycolysis (Levine and Puzio-Kuter 2010; Feron 2009) and is considered an important step in carcinogenesis (Schwartz et al. 2017; Hanahan and Weinberg 2011). Mutations in well-known oncogenes *KRAS*, *PIK3CA*, and *BRAF* have been reported to drive metabolic reprogramming towards the Warburg-effect (Levine and Puzio-Kuter 2010; Kimmelman 2015; Hutton et al. 2016; Jiang et al. 2018). Furthermore, we have previously shown in CRC that DNA mismatch repair deficiency (dMMR), a surrogate for microsatellite instability (MSI), was associated with the Warburg-effect (Offermans et al. 2021).

MSI and *KRAS*, *PIK3CA*, and *BRAF* mutations (*KRAS*_mut_*, PIK3CA*_mut_, *BRAF*_mut_, respectively) are common molecular features in CRC (Li et al. 2020; Haluska et al. 2007; Boland and Goel 2010). Associations between energy balance-related factors (i.e. BMI, waist circumference, physical activity) and risk of CRC in relation to *KRAS*_mut_, *BRAF*_mut_*,* and MSI/MMR status have been reported previously (Carr et al. 2018, 2020; Myte et al. 2019; Brändstedt et al. 2013, 2014; Slattery et al. 2000, 2001, 2007; Hughes et al. 2012; Campbell et al. 2010; Hoffmeister et al. 2013; Hanyuda et al. 2016). However, results thus far are inconsistent. To the best of our knowledge, there are no studies that have investigated associations between energy balance-related factors and risk of CRC in relation to *PIK3CA*_mut_ status.

The aim of the current study was to investigate the associations of BMI, lower body clothing-size (as a proxy for waist circumference), and physical activity with risk of CRC subgroups based on *KRAS*_mut_*, PIK3CA*_mut_, *BRAF*_mut_*,* and MMR status. First, we compared CRC subgroups based on a combination of these molecular features: I) all-wild-type + pMMR — cases wild-type for all genes (*KRAS*, *PIK3CA*, and *BRAF*) and MMR-proficient (pMMR); II) any-mutation/dMMR — cases with a mutation in any of the genes (*KRAS*, *PIK3CA*, and/or *BRAF*) and/or dMMR. Second, we investigated subgroups of these molecular features individually: *KRAS*_mut_, *PIK3CA*_mut_, *BRAF*_mut_, and dMMR. The all-wild-type+pMMR subgroup served as the reference group for all other subgroups.

We hypothesized that associations between energy balance-related factors and risk of CRC differ between subgroups based on *KRAS*_mut_, *PIK3CA*_mut_, *BRAF*_mut_, and MMR status, which could indicate involvement of the Warburg-effect in etiological associations. We reasoned that associations with subgroups of individual molecular features (*KRAS*_mut_, *PIK3CA*_mut_, *BRAF*_mut_, or dMMR) and/or with the any-mutation/dMMR subgroup, but not the all-wild-type + pMMR subgroup, give an indication of involvement of the Warburg-effect in the etiological pathway between the exposure of interest and CRC.

## Methods

### Design and study population

**Fig. 1:**
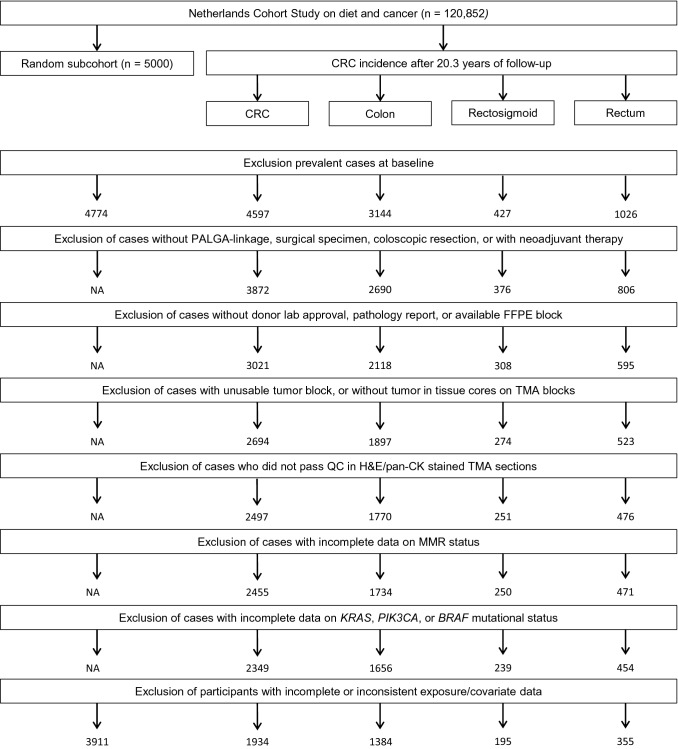
Flow diagram of the number of CRC cases and subcohort members; NLCS, 1986–2006. *CRC *colorectal cancer; *NA* not applicable; *PALGA* Dutch Pathology Registry; *FFPE* formalin-fixed paraffin-embedded; *TMA* tissue microarray; *QC* quality control; *H&E* Hematoxylin & Eosin; *pan-CK* pan-cytokeratin; *MMR* mismatch repair

Data from the Netherlands Cohort Study (NLCS), a large prospective cohort study, was used. At baseline (1986), 120,852 subjects aged 55–69 years completed a mailed, self-administered questionnaire on cancer risk factors (Brandt et al. 1990a). By completing and returning the questionnaire, participants agreed to participate in the study. The NLCS was approved by institutional review boards from Maastricht University and the Netherlands Organization for Applied Scientific Research. Ethical approval was obtained from the Medical Ethical Committee of Maastricht University Medical Center + . For data processing and analysis, a case-cohort approach was used (Prentice 1986). A subcohort (*n* = 5000) was randomly sampled from the total cohort immediately after baseline, and accumulated person-years were estimated from this subcohort. Vital status information of subcohort members was obtained biennially by active follow-up and by linkage with municipal population registries. Incident cancer cases from the total cohort were detected through annual record linkage with the Netherlands Cancer Registry and PALGA, the nationwide Dutch Pathology Registry (Brandt et al. 1990b), covering 20.3 years of follow-up (September 17, 1986 until January 1, 2007). Completeness of cancer follow-up by the Netherlands Cancer Registry and PALGA was estimated to be over 96% (Goldbohm et al. 1994). After excluding cases and subcohort members who reported a history of cancer (except skin cancer) at baseline, a total of 4,597 incident CRC cases and 4,774 subcohort members were available (Fig. 1). As described previously (Jenniskens et al. 2021a), formalin-fixed paraffin-embedded (FFPE) tissue blocks from primary tumor and matched normal colon tissue from 3,872 CRC cases were requested from participating laboratories as part of the Rainbow-TMA project during 2012–2017. Tissue blocks from 3,021 CRC cases were successfully collected from 43 pathology laboratories throughout the Netherlands (78% retrieval rate) (Fig. 1).

### Mismatch repair status

From the FFPE blocks, 78 tissue microarrays (TMAs) were constructed sampling three 0.6 mm tumor cores from 2,694 CRC cases (Fig. 1). Information on TMA construction has been published previously (Jenniskens et al. 2021a). Five μm thick sections were cut from all TMA blocks, stained with Hematoxylin & Eosin (H&E) according to a standard protocol, and subjected to immunohistochemistry (IHC) using an automated immunostainer (DAKO Autostainer Link 48, Glostrup, Denmark). MMR status, a surrogate for the presence or absence of MSI, was assessed using IHC staining of MLH1 and MSH2 as described previously (Offermans et al. 2021). All TMA sections were scanned using an Aperio scanner (Leica Microsystems, Milton Keynes, UK) at 40 × magnification at the University of Leeds (UK) Scanning Facility or at the Department of Pathology, Aachen University Hospital (Germany).

H&E-stained TMA sections combined with pan-cytokeratin stained sections (if necessary) were reviewed to confirm presence of adenocarcinoma for each core. Requiring at least one core per case with adenocarcinoma, 2497 cases passed quality control (Fig. 1). IHC scoring of MLH1 and MSH2 was performed according to the protocol published by Richman et al. (2016) by an experienced histopathologist (HG) as well as by three trained (Jenniskens et al. 2021b) non-pathologists (G.E. Fazzi: histology technician; K. Offermans: PhD student; J.C.A. Jenniskens: PhD student). Tumors with complete loss of either MLH1 or MSH2 expression were classified as MMR-deficient (dMMR), and those expressing both MLH1 and MSH2 were classified as MMR-proficient (pMMR). MMR status information was available for 2,455 CRC cases (Fig. 1).

### DNA isolation and mutation detection

For DNA extraction, two 20 µm thick sections were cut from FFPE blocks containing primary tumor. Sections were deparaffinized manually using the Buffer ATL (Cat. No. 939011, Qiagen, Hilden, Germany), Proteinase K (Cat. No. 19131, Qiagen), and the Deparaffinization Solution (Cat. No. 19093, Qiagen), using an adapted version of the manufacturer’s protocol (Supplementary Methods). The QIAsymphony® DSP DNA Mini Kit (Cat. No. 937236, Qiagen) and the QIAsymphony® (Qiagen) instrument were used for DNA isolation following the manufacturer’s protocol (Tissue_HC_200 protocol). The Quantus™ Fluorometer (Promega, Madison, WI, USA) with a QuantiFluor® dsDNA system (Promega) was used to determine the double-stranded DNA concentrations. Mutations in tumor DNA were analyzed at Institut für Immunologie und Genetik (Kaiserslautern, Germany) with the ColoCarta panel (Agena Bioscience, Hamburg), which screens for 32 mutations in 6 genes (*BRAF, HRAS, KRAS*, *MET, NRAS, PIK3CA*; see Supplementary Table S1 for specific mutations) using Matrix Assisted Laser Desorption Ionization-Time of Flight (MALDI-TOF) mass spectrometry. To ensure valid mutation information, the following cut-offs were used: *Z*-score ≥ 4.00; spectrum quality ≥ 0.750; typer peak probability ≥ 0.850; primer extension rate cut-off ≥ 0.200. Detection of mutations at a frequency of ≥ 7.5% for any of the alleles was considered evidence of a mutation in the corresponding gene. A failed reaction at a single nucleotide position resulted in missing data for the corresponding gene status only if the reactions at all other positions were wild-type.

No mutations were observed in *HRAS*, and *NRAS* mutations were found in a total of 86 cases. *NRAS* mutations were not included in the current analyses as after stratification on sex and tumor location, subgroups would have less than 50 cases (range 10–42 cases). This would have led to empty cells or cells with less than five cases for models based on categories of exposures. Complete information on *KRAS*, *PIK3CA*, and *BRAF* mutation status as well as MMR status was available for 2,349 CRC cases (Fig. 1). Supplementary Table S2 shows baseline characteristics of CRC cases by availability of mutation and MMR status.

### Subgroups of molecular features

The following subgroups were used for statistical analyses: (I) all-wild-type + pMMR — cases wild-type for all genes (*KRAS*, *PIK3CA*, and *BRAF*) and pMMR; (II) any-mutation/dMMR — cases with a mutation in any of the genes (*KRAS*, *PIK3CA*, and *BRAF*) and/or dMMR; (III) *KRAS*_mut _— cases with a (non-exclusive) *KRAS* mutation; (IV) *BRAF*_mut_; (V) *PIK3CA*_mut_; and (VI) dMMR. Note: subgroups of individual mutation and MMR status might overlap since multiple mutations and/or dMMR can occur within the same tumor.

### Energy balance-related factors

Baseline questionnaires provided information on anthropometry, physical activity, diet, and other risk factors (Brandt et al. 1990a). BMI at baseline (kg/m^2^) was calculated using baseline weight (kg) divided by height squared (m^2^). Lower body clothing-size (trouser/skirt) was used as a proxy for waist circumference (Hughes et al. 2009). Non-occupational physical activity included leisure activities like walking, cycling, or doing sports, as described in more detail previously (Simons et al. 2013). Occupational energy expenditure and sitting time were estimated for the longest held job, which was self-reported at baseline. Jobs were classified as low, moderate, or high activity, as described previously (Simons et al. 2013). Energy expenditure was classified as < 8, 8–12, and > 12 kJ/minute, and sitting time as sitting for > 6, 2–6, and < 2 working hours/day. Data on occupational physical activity were only available for the subcohort and for cases until 17.3 years of follow-up, since funding for later data-entry and classification of occupations was unavailable. Furthermore, we did not analyze occupational physical activity measures in women because many did not have paid jobs (Simons et al. 2013).

### Statistical analyses

After exclusion of participants with incomplete or inconsistent data on exposure variables or confounders, 3911 subcohort members and 1934 CRC cases were available for analyses (Fig. 1). Descriptive statistics and frequency distributions were calculated for subgroups based on molecular features and cohort characteristics. Differences of molecular features between men and women and between colon and rectum were evaluated using Chi-square. Associations between energy balance-related factors and CRC subgroups based on molecular features were investigated stratified on sex and tumor location. Cox proportional hazard models were used to estimate hazard ratios (HRs) and 95% confidence intervals (CIs) for the associations between CRC and BMI (according to sex-specific quartiles, and per 5 kg/m^2^ increase), clothing-size (according to sex-specific quartiles, and per 2 sizes increase), non-occupational physical activity (in categories of < 30, 30–60, 60–90, > 90 min per day, and per 30 min/day increase), and, for men, occupational physical activity (energy expenditure in categories of < 8, 8–12, > 12 kJ/minute; sitting time in categories of > 6, 2–6, and < 2 working hours/day). Standard errors of the HRs were estimated using the Huber-White sandwich estimator to account for additional variance introduced by sampling from the cohort (Lin and Wei 1989). The proportional hazard assumption was tested using the scaled Schoenfeld residuals (Schoenfeld 1982) and by introducing time-covariate interactions into the models.

All multivariable models were adjusted for age, family history of CRC (yes/no), alcohol intake (0; 0.1–4; 5–14; > 15 g/day), energy intake at baseline (kcal/day), red meat consumption (g/day), and processed meat consumption (g/day), as used previously (Jenniskens et al. 2021a). In addition, BMI and clothing-size models were adjusted for non-occupational physical activity (minutes/day), and BMI models for height (cm). All physical activity models were adjusted for BMI. Moreover, an additional analysis was conducted with mutual adjustment for clothing-size and BMI, where clothing-size adjusted for BMI represents a proxy for abdominal fatness, and BMI adjusted for clothing-size a proxy for subcutaneous fatness (Hughes et al. 2009; Janssen et al. 2002). Sensitivity analyses were performed excluding the first two years of follow-up.

Heterogeneity in associations between energy balance-related factors and CRC subgroups based on molecular features was evaluated using an adapted version of the competing risks procedure in Stata developed specifically for the case-cohort design (Vogel et al. 2008). The original procedure assumes independence of both estimated HRs, which underestimates the standard error and thus overestimates the *p*-values for their difference. Therefore, the *p*-values and associated CIs were estimated based on a bootstrapping method developed specifically for the case-cohort design (Wacholder et al. 1989). Each bootstrap analysis was based on 1000 replications. The all-wild-type + pMMR subgroup was the reference group for heterogeneity tests of all subgroups. Since our analyses were hypothesis-driven and exposures reflect different aspects of energy balance, we did not correct for multiple testing. All analyses were conducted in Stata Statistical Software: Release 15 (StataCorp., 2017, College Station, TX).

## Results

### Frequencies of molecular features

**Fig. 2:**
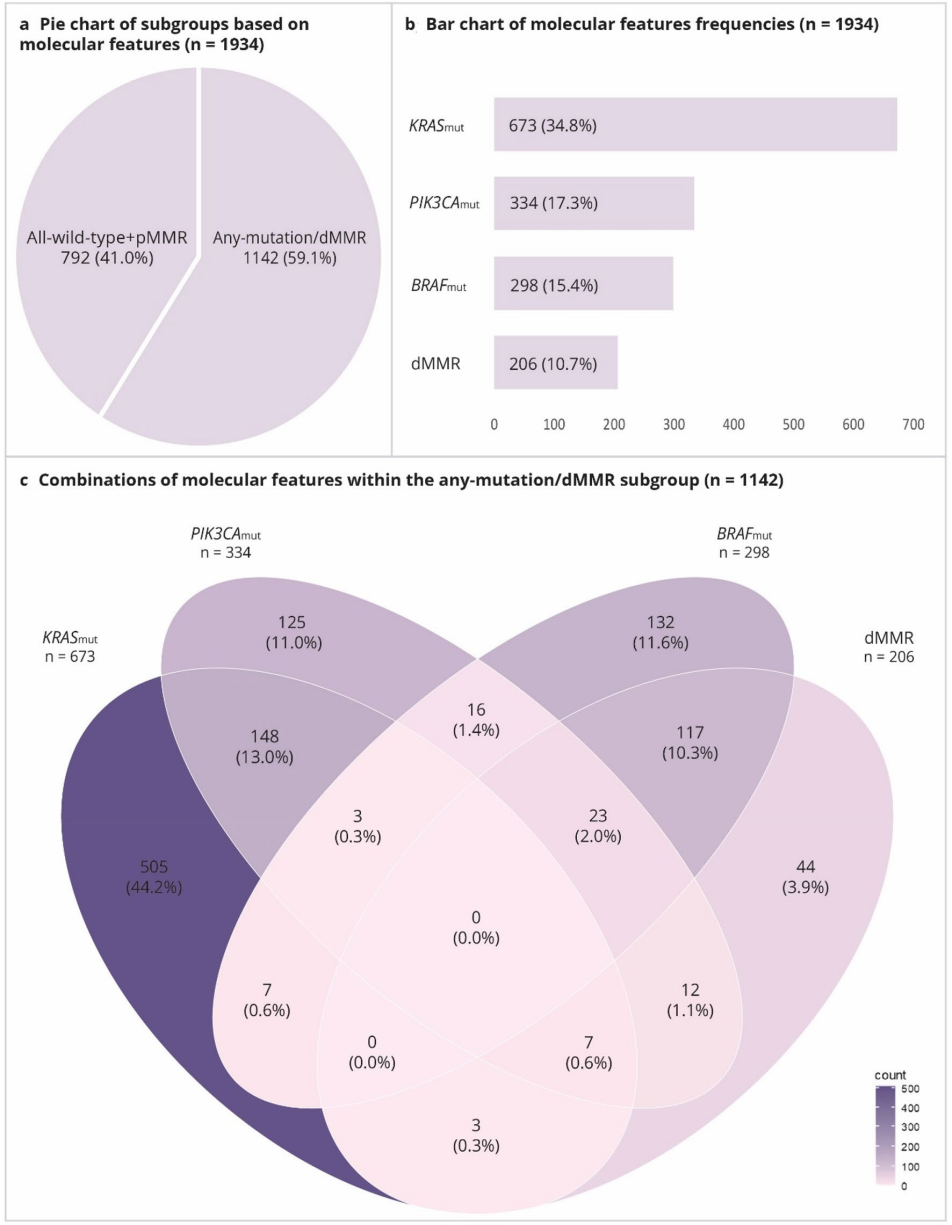
Graphical presentation of *KRAS*_mut_, *PIK3CA*_mut_, *BRAF*_mut_, and MMR status in CRC cases from the NLCS. **a** Pie chart showing the distribution of the all-wild-type + pMMR and any-mutation/dMMR subgroups (based on all CRC cases; *n* = 1934). **b** Bar chart showing frequencies of *KRAS*_mut_, *PIK3CA*_mut_, *BRAF*_mut_, and dMMR (based on all CRC cases; *n* = 1934). **c** Venn diagram showing combinations of *KRAS*_mut_, *PIK3CA*_mut_, *BRAF*_mut_, and dMMR (based on any-mutation/dMMR subgroup; *n* = 1142). The color intensity indicates the frequency: a darker color indicates more cases; a lighter color indicates fewer cases. *d/pMMR* mismatch repair deficiency/proficiency; *mut* mutation; *CRC* colorectal cancer; *NLCS* Netherlands Cohort Study

In total, 1142 (59.1%) tumors had a mutation in at least one of the genes (*KRAS*, *PIK3CA*, or *BRAF*) and/or were classified as dMMR (Table 1, Fig. 2a). The overall frequency of mutations and/or presence of dMMR was higher in women compared to men (66.4% vs 53.6%, respectively; *p*-value: < 0.001), and higher in tumors located in the colon compared to the rectum (64.7% vs 43.9%, respectively; *p*-value: < 0.001) (Table 1).

**Table 1:** Frequencies^a^ [*n* (%)] of subgroups based on mutation and MMR status in CRC cases, by tumor location and sex; NLCS, 1986–2006

	CRC			*p*^d^	Colon			Rectum			*p*^e^
	Total *n* = 1934	Men *n* = 1113	Women *n* = 821		Total *n* = 1384	Men *n* = 754	Women *n* = 630	Total *n* = 355	Men *n* = 224	Women *n* = 131	
All-wild-type + pMMR^b^	792 (41.0)	516 (46.4)	276 (33.6)	< 0.001	488 (35.3)	309 (41.0)	179 (28.4)	199 (56.1)	135 (60.3)	64 (48.9)	< 0.001
Any-mutation/dMMR^c^	1142 (59.1)	597 (53.6)	545 (66.4)		896 (64.7)	445 (59.0)	451 (71.6)	156 (43.9)	89 (39.7)	67 (51.2)	
* KRAS*_mut_	673 (34.8)	376 (33.8)	297 (36.2)	0.275	478 (34.5)	256 (34.0)	222 (35.2)	123 (34.7)	68 (30.4)	55 (42.0)	0.969
* PIK3CA*_mut_	334 (17.3)	196 (17.6)	138 (16.8)	0.645	266 (19.2)	150 (19.9)	116 (18.4)	45 (12.7)	30 (13.4)	15 (11.5)	0.004
* BRAF*_mut_	298 (15.4)	117 (10.5)	181 (22.1)	< 0.001	278 (20.1)	105 (13.9)	173 (27.5)	14 (3.9)	8 (3.6)	6 (4.6)	< 0.001
dMMR	206 (10.7)	72 (6.5)	134 (16.3)	< 0.001	201 (14.5)	70 (9.3)	131 (20.8)	3 (0.9)	1 (0.5)	2 (1.5)	< 0.001

*(d/p)MMR* mismatch repair (deficient/proficient); *CRC* colorectal cancer; *NLCS* Netherlands Cohort Study; *mut* mutated

^a^Percentages might not add up because multiple molecular characteristics (e.g. *BRAF* mutation and MMR deficiency) can occur per individual

^b^This group excludes cases with mutations in any of the genes (*KRAS*, *PIK3CA*, or *BRAF*), as well as MMR deficient cases

^c^This group includes cases with mutations in any of the genes (*KRAS*, *PIK3CA*, or *BRAF*) and/or cases that are MMR deficient

^d^Difference between men and women, based on total CRC, evaluated using Chi-square

^e^Difference between colon and rectum, based on men and women combined, evaluated using Chi-square

*KRAS*_mut_-tumors were observed in 673 (34.8%) cases, *PIK3CA*_mut_-tumors in 334 (17.3%) cases, *BRAF*_mut_-tumors in 298 (15.4%) cases, and dMMR-tumors in 206 (10.7%) cases (Table 1, Fig. 2b). The frequency of *BRAF*_mut_-tumors and dMMR-tumors was higher in women compared to men (*BRAF*_mut_: 22.1 vs 10.5%, *p*-value: < 0.001; dMMR: 16.3% vs 6.5%, *p*-value: < 0.001, respectively). *PIK3CA*_mut_-, *BRAF*_mut_-, and dMMR-tumors were more often observed in colon compared to rectum (*PIK3CA*_mut_: 19.2% vs 12.7%, *p*-value: 0.004; *BRAF*_mut_: 20.1% vs 3.9%, *p*-value: < 0.001; dMMR: 14.5% vs 0.9%, *p*-value: < 0.001, respectively) (Table 1).

Within the any-mutation/dMMR subgroup, exclusive *KRAS*_mut_-tumors were observed in 505 (44.2%), exclusive *PIK3CA*_mut_-tumors in 125 (11.0%), exclusive *BRAF*_mut_-tumors in 132 (11.6%), and exclusive dMMR-tumors in 44 (3.9%) cases (Fig. 2c). Combinations of *KRAS*_mut_ and *PIK3CA*_mut_ and of *BRAF*_mut_ and dMMR were most common (13.0% and 10.3%, respectively). Other combinations of mutations and/or dMMR were relatively rare (i.e. < 5%) (Fig. 2c).

### Cohort characteristics in subgroups based on molecular features

Information on cohort characteristics of CRC cases, overall and according to subgroups based on molecular features, is provided in Table 2. Cases in the any-mutation/dMMR subgroup were older than those in the all-wild-type + pMMR subgroup. Furthermore, cases in the any-mutation/dMMR subgroup were more often overweight compared to those in the all-wild-type + pMMR subgroup, with the exception of men with colon cancer. In general, overweight was most frequently observed amongst cases with *KRAS*_mut_- and/or *PIK3CA*_mut_-tumors. Similarly, the any-mutation/dMMR subgroup showed a larger mean clothing-size compared to the all-wild-type + pMMR subgroup, with the exception of men with colon cancer. The mean clothing-size was largest for the *KRAS*_mut_ subgroup, again with the exception of men with colon cancer. Non-occupational physical activity was higher amongst the all-wild-type + pMMR subgroup than amongst the any-mutation/dMMR subgroup, with the exception of women with rectal cancer. In men, cases with a *PIK3CA*_mut_-tumor in the colon were least physically active, whereas in women cases with dMMR- or *BRAF*_mut_-tumors in the colon were least physically active. Colon cancer cases in the any-mutation/dMMR subgroup showed a higher occupational energy expenditure than those in the all-wild-type + pMMR subgroup. In particular, dMMR colon cancer cases showed the highest occupational energy expenditure and lowest occupational sitting time. In contrast, rectal cancer cases in the any-mutation/dMMR subgroup showed lower occupational energy expenditure compared to those in the all-wild-type + pMMR subgroup.

**Table 2:** Characteristics [mean (SD) or %] of CRC cases in subgroups based on mutation and MMR status, by sex and tumor location; NLCS, 1986–2006

	Total	Wild-type + pMMR^d^	Any-mutation/dMMR^e^	*KRAS*_mut_	*PIK3CA*_mut_^f^	*BRAF*_mut_^f^	dMMR^f^
*Men—colon*							
*N*	754	309	445	256	150	105	70
Age (years)	61.6 (4.2)	61.2 (4.2)	61.9 (4.2)	62.0 (4.2)	61.5 (4.3)	62.2 (4.1)	62.7 (4.1)
Overweight/obesity^a^ (%)	52.4	52.8	52.1	52.7	56.7	48.6	54.3
Clothing size^b^	52.2 (2.6)	52.2 (2.5)	52.2 (2.7)	52.1 (2.7)	52.1 (2.7)	52.2 (2.9)	52.3 (2.8)
Non-occupational PA > 60 min/day (%)	50.4	53.7	48.1	51.6	42.0	49.5	55.7
Occ. energy expenditure (> 12 kJ/min)^c^	11.6	8.8	13.4	13.2	13.9	11.2	17.0
Occ. sitting time (< 2 h/day)^c^	23.2	23.5	22.9	23.5	27.1	20.2	28.8
*Men—rectum*							
*N*	224	135	89	68	30	8	1
Age (years)	60.8 (3.9)	60.4 (4.0)	61.4 (3.9)	61.7 (3.9)			
Overweight/obesity^a^ (%)	48.2	46.7	50.6	52.9			
Clothing size^b^	51.7 (2.5)	51.6 (2.3)	51.8 (2.8)	52.3 (2.8)			
Non-occupational PA > 60 min/day (%)	59.4	60.0	58.4	54.4			
Occ. energy expenditure (> 12 kJ/min)^c^	11.0	12.1	9.3	8.8			
Occ. sitting time (< 2 h/day)^c^	30.4	31.0	29.3	29.8			
*Women—colon*							
*N*	630	179	451	222	116	173	131
Age (years)	62.0 (4.1)	61.1 (3.9)	62.3 (4.1)	62.2 (4.1)	62.2 (4.2)	62.6 (4.1)	62.3 (4.0)
Overweight/obesity^a^ (%)	44.8	39.7	46.8	52.7	49.1	43.4	38.2
Clothing size^b^	43.6 (3.4)	43.4 (4.1)	43.6 (3.0)	43.9 (3.2)	43.5 (2.9)	43.6 (2.8)	43.3 (3.0)
Non-occupational PA > 60 min/day (%)	41.8	46.4	39.9	40.1	38.8	38.7	41.2
*Women—rectum*							
*N*	131	64	67	55	15	6	2
Age (years)	61.5 (4.2)	60.9 (4.3)	62.0 (4.0)	61.7 (4.0)			
Overweight/obesity^a^ (%)	49.6	48.4	50.8	52.7			
Clothing size^b^	43.5 (2.7)	43.3 (2.7)	43.8 (2.7)	43.9 (2.8)			
Non-occupational PA > 60 min/day (%)	42.8	37.5	47.8	47.3			

*SD* standard deviation; *CRC* colorectal cancer; *(d/p)MMR* mismatch repair (deficient/proficient); *NLCS* Netherlands Cohort Study; *PA* physical activity.; *Occ* occupational

^a^Body mass index ≥ 25

^b^Lower body clothing size. Based on fewer participants due to extra missings

^c^Based on fewer participants due to shorter follow-up (17.3 years), only available for men

^d^This group excludes cases with mutations in any of the genes (*KRAS*, *PIK3CA*, or *BRAF*), as well as MMR deficient cases

^e^This group includes cases with mutations in any of the genes (*KRAS*, *PIK3CA*, or *BRAF*) and/or cases that are MMR deficient

^f^Analyses for subgroups with < 50 cases were not performed

### Associations of energy balance-related factors and CRC subgroups based on molecular features

Multivariable-adjusted Cox-regression models on energy balance-related factors and risk of CRC subgroups based on molecular features are shown in Tables 3, 4, 5, and 6. Age-adjusted Cox-regression models are shown in Supplementary Tables S3–S6. Results of associations between energy balance-related factors and risk of CRC wild-type and MMR-proficient subgroups separately are additionally presented in Supplementary Tables S7–S8. Age was included as a time-varying covariate in all models, because of violation of the proportional hazards assumption.

**Table 3:** Multivariable-adjusted HRs^a^ and 95%-CIs for associations between adiposity measures and CRC in subgroups based on mutation and MMR status, by sex and tumor location; NLCS, 1986–2006

	Person-years at risk	Total		Wild-type + pMMR^b^		Any-mutation/dMMR^c^		*p*-het
		*n*_cases_	HR (95% CI)	*n*_cases_	HR (95% CI)	*n*_cases_	HR (95% CI)	
**BMI quartiles** **(kg**/**m**^**2**^)**: range (median)**								
*Men—colon*								
< 23.4 (22.2)	7993	167	1.00 (ref.)	66	1.00 (ref.)	101	1.00 (ref.)	
23.4–24.9 (24.2)	8343	188	1.06 (0.82–1.37)	79	1.10 (0.76–1.57)	109	1.04 (0.77–1.42)	
25.0–26.6 (25.7)	7683	200	1.21 (0.93–1.56)	77	1.13 (0.78–1.64)	123	1.26 (0.92–1.72)	
> 26.6 (27.8)	7003	199	1.41 (1.09–1.83)	87	1.47 (1.03–2.11)	112	1.37 (0.99–1.88)	0.710
*p*-trend			0.005		0.038		0.027	
per 5 kg/m^2^	31,022	754	1.30 (1.12–1.51)	309	1.34 (1.08–1.67)	445	1.28 (1.07–1.53)	0.454
*Men—rectum*								
< 23.4 (22.2)	7993	58	1.00 (ref.)	34	1.00 (ref.)	24	1.00 (ref.)	
23.4–24.9 (24.2)	8343	54	0.86 (0.57–1.28)	35	0.95 (0.58–1.57)	19	0.73 (0.39–1.37)	
25.0–26.6 (25.7)	7683	65	1.15 (0.78–1.69)	42	1.29 (0.80–2.09)	23	0.95 (0.51–1.74)	
> 26.6 (27.8)	7003	47	0.93 (0.61–1.43)	24	0.82 (0.47–1.44)	23	1.09 (0.59–2.01)	0.458
*p*-trend			0.851		0.870		0.636	
per 5 kg/m^2^	31,022	224	1.02 (0.81–1.28)	135	0.95 (0.71–1.26)	89	1.14 (0.80–1.62)	0.387
*Women—colon*								
< 22.8 (21.5)	9014	181	1.00 (ref.)	56	1.00 (ref.)	125	1.00 (ref.)	
22.8–24.7 (23.8)	8914	146	0.81 (0.63–1.05)	43	0.78 (0.51–1.19)	103	0.83 (0.62–1.11)	
24.8–27.0 (25.7)	8141	147	0.92 (0.71–1.20)	36	0.73 (0.47–1.16)	111	1.01 (0.75–1.36)	
> 27.0 (29.2)	8158	156	1.01 (0.77–1.31)	44	0.91 (0.59–1.41)	112	1.05 (0.78–1.43)	0.601
*p*-trend			0.805		0.595		0.516	
per 5 kg/m^2^	34,228	630	1.04 (0.92–1.18)	179	0.88 (0.69–1.11)	451	1.11 (0.96–1.27)	0.081
*Women—rectum*								
< 22.8 (21.5)	9014	37	1.00 (ref.)	16	1.00 (ref.)	21	1.00 (ref.)	
22.8–24.7 (23.8)	8914	26	0.69 (0.41–1.17)	15	0.90 (0.43–1.89)	11	0.53 (0.25–1.13)	
24.8–27.0 (25.7)	8141	32	0.91 (0.55–1.53)	18	1.16 (0.58–2.34)	14	0.75 (0.36–1.56)	
> 27.0 (29.2)	8158	36	1.04 (0.63–1.72)	15	0.93 (0.45–1.90)	21	1.20 (0.61–2.38)	0.491
*p*-trend			0.691		0.973		0.548	
per 5 kg/m^2^	34,228	131	1.08 (0.86–1.34)	64	1.05 (0.77–1.42)	67	1.12 (0.82–1.53)	0.872
**Clothing size: range (median)**								
*Men—colon*								
≤ 50 (50)	10,903	211	1.00 (ref.)	90	1.00 (ref.)	121	1.00 (ref.)	
52 (52)	9750	247	1.30 (1.03–1.62)	104	1.29 (0.94–1.77)	143	1.30 (0.98–1.71)	
54 (54)	5156	136	1.36 (1.04–1.77)	53	1.26 (0.86–1.84)	83	1.43 (1.03–1.98)	
≥ 56 (56)	2619	90	1.80 (1.31–2.46)	39	1.87 (1.22–2.86)	51	1.75 (1.19–2.57)	0.897
*p*-trend			< 0.001		0.008		0.002	
per 2 sizes	28,428	684	1.33 (1.16–1.52)	286	1.34 (1.12–1.61)	398	1.32 (1.11–1.55)	0.983
*Men—rectum*								
≤ 50 (50)	10,903	78	1.00 (ref.)	46	1.00 (ref.)	32	1.00 (ref.)	
52 (52)	9750	69	1.00 (0.70–1.41)	46	1.12 (0.73–1.72)	23	0.80 (0.46–1.42)	
54 (54)	5156	43	1.20 (0.80–1.80)	28	1.17 (0.70–1.96)	18	1.23 (0.67–2.25)	
≥ 56 (56)	2619	16	0.90 (0.51–1.59)	7	0.67 (0.29–1.51)	9	1.23 (0.57–2.66)	0.454
*p*-trend			0.801		0.760		0.470	
per 2 sizes	28,428	206	0.98 (0.81–1.20)	124	0.95 (0.75–1.21)	82	1.02 (0.74–1.41)	0.711
*Women—colon*								
≤ 40 (40)	6574	128	1.00 (ref.)	46	1.00 (ref.)	82	1.00 (ref.)	
42 (42)	8582	150	0.88 (0.67–1.17)	34	0.58 (0.36–0.93)	116	1.05 (0.76–1.46)	
44 (44)	9270	159	0.83 (0.63–1.10)	48	0.74 (0.48–1.15)	111	0.89 (0.64–1.23)	
≥ 46 (46)	9454	182	0.95 (0.72–1.26)	50	0.78 (0.50–1.20)	132	1.06 (0.77–1.46)	0.104
*p*-trend			0.764		0.537		0.979	
per 2 sizes	33,880	619	1.08 (0.95–1.24)	178	1.07 (0.82–1.40)	441	1.09 (0.94–1.26)	0.759
*Women—rectum*								
≤ 40 (40)	6574	23	1.00 (ref.)	11	1.00 (ref.)	12	1.00 (ref.)	
42 (42)	8582	30	0.94 (0.53–1.67)	17	1.13 (0.52–2.49)	13	0.77 (0.34–1.73)	
44 (44)	9270	35	1.01 (0.58–1.75)	20	1.28 (0.62–2.65)	15	0.79 (0.35–1.77)	
≥ 46 (46)	9454	42	1.14 (0.66–1.97)	16	0.96 (0.44–2.08)	26	1.31 (0.62–2.78)	0.319
*p*-trend			0.532		0.941		0.355	
per 2 sizes	33,880	130	0.99 (0.80–1.23)	64	0.96 (0.71–1.28)	66	1.02 (0.76–1.39)	0.562

*HR* hazard ratio; *CI* confidence interval; *CRC* colorectal cancer; *(d/p)MMR* mismatch repair (deficient/proficient); *NLCS* Netherlands Cohort Study; *BMI* body mass index; *p*-het *p*-heterogeneity

^a^Hazard ratios were adjusted for age (years; continuous), non-occupational physical activity (min/day; continuous), total energy intake (kcal/day; continuous), family history of CRC (yes/no), alcohol consumption (0; 0.1–4; 5–14; > 15 g/day), processed meat intake (g/day; continuous), red meat intake (g/day; continuous). Age was included as a time-varying covariate. BMI models were additionally adjusted for height (cm; continuous)

^b^This group excludes cases with mutations in any of the genes (*KRAS*, *PIK3CA*, or *BRAF*), as well as MMR deficient cases

^c^This group includes cases with mutations in any of the genes (*KRAS*, *PIK3CA*, or *BRAF*) and/or cases that are MMR deficient

**Table 4:** Multivariable-adjusted HRs^a^ and 95%-CIs for associations between adiposity measures and CRC for individual mutations and MMR status, by sex and tumor location; NLCS, 1986–2006

	Person-years at risk	*KRAS*_mut_		*PIK3CA*_mut_^b^		*BRAF*_mut_^b^		dMM﻿R^b^	
		*n*_cases_	HR (95% CI)	*n*_cases_	HR (95% CI)	*n*_cases_	HR (95% CI)	*n*_cases_	HR (95% CI)
**BMI quartiles (kg/m**^**2**^**): range (median)**									
*Men—colon*									
< 23.4 (22.2)	7993	61	1.00 (ref.)	27	1.00 (ref.)	25	1.00 (ref.)	19	1.00 (ref.)
23.4–24.9 (24.2)	8343	58	0.89 (0.60–1.32)	38	1.37 (0.81–2.30)	28	1.11 (0.62–1.96)	13	0.68 (0.33–1.42)
25.0–26.6 (25.7)	7683	69	1.12 (0.76–1.65)	42	1.66 (0.98–2.81)	29	1.22 (0.68–2.20)	21	1.21 (0.63–2.34)
> 26.6 (27.8)	7003	68	1.30 (0.88–1.92)	43	1.97 (1.17–3.32)	23	1.14 (0.61–2.14)	17	1.17 (0.59–2.31)
*p*-trend			0.112		0.007		0.603		0.378
per 5 kg/m^2^	31,022	256	1.25 (1.00–1.57)	150	1.38 (1.05–1.82)	105	1.23 (0.87–1.72)	70	1.51 (1.01–2.26)
*Men—rectum*									
< 23.4 (22.2)	7993	18	1.00 (ref.)						
23.4–24.9 (24.2)	8343	13	0.67 (0.31–1.44)						
25.0–26.6 (25.7)	7683	19	1.06 (0.53–2.15)						
> 26.6 (27.8)	7003	18	1.21 (0.59–2.47)						
*p*-trend			0.415						
per 5 kg/m^2^	31,022	68	1.17 (0.79–1.73)						
*Women—colon*									
< 22.8 (21.5)	9014	52	1.00 (ref.)	30	1.00 (ref.)	53	1.00 (ref.)	43	1.00 (ref.)
22.8–24.7 (23.8)	8914	46	0.88 (0.58–1.35)	27	0.89 (0.52–1.53)	40	0.76 (0.49–1.19)	34	0.78 (0.49–1.26)
24.8–27.0 (25.7)	8141	65	1.48 (0.99–2.20)	30	1.08 (0.64–1.84)	39	0.82 (0.52–1.29)	24	0.61 (0.36–1.03)
> 27.0 (29.2)	8158	59	1.37 (0.90–2.08)	29	1.01 (0.60–1.73)	41	0.90 (0.57–1.41)	30	0.78 (0.48–1.30)
*p*-trend			0.031		0.798		0.678		0.221
per 5 kg/m^2^	34,228	222	1.31 (1.10–1.57)*	116	1.09 (0.84–1.42)	173	0.99 (0.81–1.22)	131	0.90 (0.70–1.15)
*Women—rectum*									
< 22.8 (21.5)	9014	18	1.00 (ref.)						
22.8–24.7 (23.8)	8914	7	0.40 (0.17–0.98)						
24.8–27.0 (25.7)	8141	9	0.57 (0.24–1.33)						
> 27.0 (29.2)	8158	21	1.46 (0.72–2.96)						
*p*-trend			0.312						
per 5 kg/m^2^	34,228	55	1.21 (0.87–1.67)						
**Clothing size: range (median)**									
*Men—colon*									
≤ 50 (50)	10,903	73	1.00 (ref.)	40	1.00 (ref.)	30	1.00 (ref.)	18	1.00 (ref.)
52 (52)	9750	84	1.26 (0.89–1.78)	52	1.45 (0.94–2.24)	29	1.04 (0.61–1.78)	23	1.37 (0.71–2.64)
54 (54)	5156	48	1.33 (0.88–2.02)	22	1.20 (0.69–2.09)	21	1.40 (0.77–2.55)	12	1.37 (0.64–2.95)
≥ 56 (56)	2619	29	1.63 (1.00–2.65)	17	1.80 (0.98–3.31)	9	1.19 (0.54–2.61)	7	1.55 (0.63–3.81)
*p*-trend			0.040		0.094		0.360		0.280
per 2 sizes	28,428	234	1.24 (1.01–1.54)	131	1.31 (1.01–1.70)	89	1.18 (0.85–1.64)	60	1.33 (0.90–1.96)
*Men—rectum*									
≤ 50 (50)	10,903	21	1.00 (ref.)						
52 (52)	9750	17	0.90 (0.45–1.77)						
54 (54)	5156	17	1.71 (0.86–3.40)						
≥ 56 (56)	2619	9	1.78 (0.78–4.06)						
*p*-trend			0.073						
per 2 sizes	28,428	64	1.17 (0.80–1.73)						
*Women—colon*									
≤ 40 (40)	6574	35	1.00 (ref.)	24	1.00 (ref.)	29	1.00 (ref.)	32	1.00 (ref.)
42 (42)	8582	54	1.13 (0.72–1.78)	25	0.75 (0.42–1.34)	47	1.23 (0.75–2.03)	35	0.82 (0.49–1.37)
44 (44)	9270	58	1.10 (0.70–1.72)	33	0.84 (0.48–1.45)	45	1.02 (0.62–1.68)	25	0.51 (0.29–0.88)
≥ 46 (46)	9454	70	1.33 (0.86–2.05)	30	0.74 (0.42–1.30)	49	1.11 (0.67–1.83)	37	0.76 (0.45–1.28)
*p*-trend			0.229		0.423		0.957		0.174
per 2 sizes	33,880	217	1.26 (1.03–1.53)	112	1.05 (0.82–1.35)	170	1.04 (0.84–1.27)	129	0.90 (0.71–1.14)
*Women—rectum*									
≤ 40 (40)	6574	10	1.00 (ref.)						
42 (42)	8582	9	0.65 (0.26–1.62)						
44 (44)	9270	12	0.76 (0.32–1.84)						
≥ 46 (46)	9454	23	1.41 (0.63–3.13)						
*p*-trend			0.243						
per 2 sizes	33,880	54	1.07 (0.77–1.51)						

*HR* hazard ratio; *CI* confidence interval; *CRC* colorectal cancer; *(d/p)MMR* mismatch repair (deficient/proficient); *NLCS* Netherlands Cohort Study; BMI body mass index; *p-het* *p*-heterogeneity

^*^Statistically significant *p*-heterogeneity, p = 0.008 (reference group: wild-type for *KRAS, PIK3CA,* and *BRAF,* and pMMR). Note: other *p*-heterogeneity tests were not statistically significant

^a^Hazard Ratios were adjusted for age (years; continuous), non-occupational physical activity (minutes/day; continuous), total energy intake (kcal/day; continuous), family history of CRC (yes/no), alcohol consumption (0; 0.1–4; 5–14; > 15 g/day), processed meat intake (g/day; continuous), red meat intake (g/day; continuous). Age was included as a time-varying covariate. BMI models were additionally adjusted for height (cm; continuous)

^b^Analyses for subgroups with < 50 cases were not performed

**Table 5:** Multivariable-adjusted HRs^a^ and 95% CIs for associations between physical activity measures and CRC in subgroups based on mutation and MMR status, by sex and tumor location; NLCS, 1986–2006

	Person-years at risk	Total		Wild-type + pMMR^b^		Any-mutation/dMMR^c^		*p*-het
		*n*_cases_	HR (95% CI)	*n*_cases_	HR (95% CI)	*n*_cases_	HR (95% CI)	
**Non-occupational physical activity (min/day): range (median)**								
*Men—colon*								
≤ 30	4997	132	1.00 (ref.)	49	1.00 (ref.)	83	1.00 (ref.)	
31–60	10,100	242	0.89 (0.69–1.16)	94	0.95 (0.64–1.39)	148	0.86 (0.63–1.17)	
61–90	6001	156	0.99 (0.75–1.32)	62	1.08 (0.71–1.63)	94	0.95 (0.67–1.33)	
> 90	9925	224	0.85 (0.65–1.11)	104	1.10 (0.76–1.60)	120	0.71 (0.51–0.97)	0.232
*p*-trend			0.356		0.402		0.050	
per 30 min/day	31,022	754	0.99 (0.95–1.03)	309	1.02 (0.97–1.07)	445	0.97 (0.92–1.02)	0.204
*Men—rectum*								
≤ 30	4997	18	1.00 (ref.)	13	1.00 (ref.)	5	1.00 (ref.)	
31–60	10,100	73	1.92 (1.12–3.30)	41	1.49 (0.78–2.85)	32	3.03 (1.16–7.89)	
61–90	6001	57	2.57 (1.47–4.47)	38	2.33 (1.21–4.47)	19	3.13 (1.15–8.52)	
> 90	9925	76	2.09 (1.22–3.59)	43	1.62 (0.85–3.08)	33	3.32 (1.28–8.60)	0.450
*p*-trend			0.012		0.104		0.033	
per 30 min/day	31,022	224	1.04 (0.98–1.09)	135	1.04 (0.96–1.11)	89	1.03 (0.96–1.11)	0.850
*Women—colon*								
≤ 30	7756	169	1.00 (ref.)	52	1.00 (ref.)	117	1.00 (ref.)	
31–60	10,923	198	0.83 (0.65–1.06)	44	0.58 (0.38–0.89)	154	0.94 (0.71–1.25)	
61–90	8000	148	0.84 (0.64–1.09)	47	0.85 (0.56–1.30)	101	0.83 (0.61–1.13)	
> 90	7550	115	0.70 (0.53–0.93)	36	0.69 (0.44–1.08)	79	0.71 (0.51–0.98)	0.145
*p*-trend			0.021		0.344		0.024	
per 30 min/day	34,228	630	0.97 (0.91–1.03)	179	0.98 (0.88–1.10)	451	0.96 (0.89–1.03)	0.623
*Women—rectum*								
≤ 30	7756	31	1.00 (ref.)	14	1.00 (ref.)	17	1.00 (ref.)	
31–60	10,923	44	1.03 (0.63–1.67)	26	1.30 (0.66–2.56)	18	0.78 (0.39–1.55)	
61–90	8000	34	1.06 (0.64–1.75)	12	0.79 (0.36–1.76)	22	1.27 (0.67–2.39)	
> 90	7550	22	0.72 (0.41–1.26)	12	0.83 (0.38–1.82)	10	0.61 (0.28–1.37)	0.214
*p*-trend			0.285		0.325		0.563	
per 30 min/day	34,228	131	1.00 (0.88–1.14)	64	1.07 (0.89–1.28)	67	0.93 (0.79–1.08)	0.206
**Occupational energy expenditure (kJ/min)**								
*Men—colon*	25,073	564		226		338		
< 8	15,144	365	1.00 (ref.)	152	1.00 (ref.)	213	1.00 (ref.)	
8–12	6368	133	0.83 (0.65–1.05)	54	0.80 (0.57–1.12)	79	0.86 (0.64–1.15)	
> 12	3561	66	0.71 (0.52–0.97)	20	0.51 (0.30–0.84)	46	0.85 (0.59–1.23)	0.201
*p*-trend			0.017		0.006		0.274	
*Men—rectum*	25,073	185		114		71		
< 8	15,144	107	1.00 (ref.)	65	1.00 (ref.)	42	1.00 (ref.)	
8–12	6368	57	1.35 (0.95–1.91)	35	1.38 (0.89–2.13)	22	1.30 (0.75–2.24)	
> 12	3561	21	0.84 (0.51–1.39)	14	0.91 (0.49–1.70)	7	0.73 (0.33–1.61)	0.956
*p*-trend			0.905		0.746		0.801	
**Occupational sitting time (h/day)**								
*Men—colon*	25,073	564		226		338		
> 6	6511	187	1.00 (ref.)	85	1.00 (ref.)	102	1.00 (ref.)	
2–6	11,617	244	0.70 (0.55–0.88)	87	0.55 (0.39–0.77)	157	0.82 (0.62–1.09)	
< 2	6944	133	0.63 (0.48–0.83)	54	0.56 (0.38–0.81)	79	0.70 (0.50–0.97)	0.102
*p*-trend			0.001		0.003		0.034	
*Men—rectum*	25,073	185		114		71		
> 6	6511	60	1.00 (ref.)	39	1.00 (ref.)	21	1.00 (ref.)	
2–6	11,617	69	0.62 (0.43–0.89)	40	0.55 (0.35–0.87)	29	0.75 (0.42–1.33)	
< 2	6944	56	0.88 (0.60–1.30)	35	0.84 (0.52–1.37)	21	0.96 (0.52–1.79)	0.730
*p*-trend			0.541		0.500		0.912	

*HR* hazard ratio; *CI* confidence interval; *CRC* colorectal cancer; *(d/p)MMR* mismatch repair (deficient/proficient); *NLCS* Netherlands Cohort Study; *p-het* *p*-heterogeneity

^a^Hazard Ratios were adjusted for age (years; continuous), BMI (kg/m^2^; continuous), total energy intake (kcal/day; continuous), family history of CRC (yes/no), alcohol consumption (0; 0.1–4; 5–14; > 15 g/day), processed meat intake (g/day; continuous), red meat intake (g/day; continuous). Age was included as a time-varying covariate

^b^This group excludes cases with mutations in any of the genes (*KRAS*, *PIK3CA*, or *BRAF*), as well as MMR deficient cases

^c^This group includes cases with mutations in any of the genes (*KRAS*, *PIK3CA*, or *BRAF*) and/or cases that are MMR deficient

**Table 6:** Multivariable-adjusted HRs^a^ and 95%-CIs for associations between physical activity measures and CRC for individual mutations and MMR status, by sex and tumor location; NLCS, 1986–2006

	Person-years at risk	*KRAS*_mut_		*PIK3CA*_mut_^b^		*BRAF*_mut_^b^		dMM﻿R^b^	
		*n*_cases_	HR (95% CI)	*n*_cases_	HR (95% CI)s	*n*_cases_	HR (95%-CI)	*n*_cases_	HR (95% CI)
**Non-occupational physical activity (min/day): range (median)**									
*Men—colon*									
≤ 30 (21.4)	4997	41	1.00 (ref.)	24	1.00 (ref.)	19	1.00 (ref.)	13	1.00 (ref.)
31–60 (42.9)	10,100	83	0.97 (0.64–1.46)	63	1.30 (0.79–2.13)	34	0.87 (0.49–1.55)	18	0.66 (0.31–1.40)
61–90 (73.6)	6001	63	1.31 (0.85–2.02)	28	0.97 (0.55–1.72)	19	0.83 (0.43–1.61)	16	1.00 (0.47–2.15)
> 90 (130.0)	9925	69	0.84 (0.55–1.28)	35	0.73 (0.42–1.26)	33	0.82 (0.46–1.48)	23	0.80 (0.39–1.64)
*p*-trend			0.575		0.041		0.548		0.925
per 30 min/day	31,022	256	0.95 (0.89–1.01)	150	0.96 (0.87–1.06)	105	1.02 (0.94–1.11)	70	1.02 (0.92–1.13)
*Men—rectum*									
≤ 30 (21.4)	4997	5	1.00 (ref.)						
31–60 (42.9)	10,100	26	2.38 (0.90–6.31)						
61–90 (73.6)	6001	16	2.66 (0.96–7.40)						
> 90 (130.0)	9925	21	2.04 (0.76–5.45)						
*p*-trend			0.372						
per 30 min/day	31,022	68	0.98 (0.89–1.08)						
*Women—colon*									
≤ 30 (19.3)	7756	55	1.00 (ref.)	36	1.00 (ref.)	47	1.00 (ref.)	32	1.00 (ref.)
31–60 (42.9)	10,923	78	1.01 (0.69–1.47)	35	0.73 (0.45–1.20)	59	0.91 (0.60–1.37)	45	1.00 (0.63–1.61)
61–90 (75.0)	8000	48	0.84 (0.56–1.27)	28	0.79 (0.47–1.32)	35	0.72 (0.45–1.16)	34	1.00 (0.60–1.67)
> 90 (115.7)	7550	41	0.80 (0.52–1.23)	17	0.51 (0.28–0.93)	32	0.70 (0.44–1.14)	20	0.64 (0.36–1.14)
*p*-trend			0.197		0.042		0.095		0.150
per 30 min/day	34,228	222	0.98 (0.90–1.08)	116	0.90 (0.77–1.04)	173	0.94 (0.84–1.05)	131	0.97 (0.85–1.09)
*Women—rectum*									
≤ 30 (19.3)	7756	14	1.00 (ref.)						
31–60 (42.9)	10,923	15	0.79 (0.37–1.68)						
61–90 (75.0)	8000	20	1.40 (0.71–2.78)						
> 90 (115.7)	7550	6	0.45 (0.17–1.18)						
*p*-trend			0.366						
per 30 min/day	34,228	55	0.86 (0.74–0.99)						
**Occupational energy expenditure (kJ/min)**									
*Men—colon*	25,073	190		114		87		58	
< 8	15,144	115	1.00 (ref.)	72	1.00 (ref.)	59	1.00 (ref.)	32	1.00 (ref.)
8–12	6368	50	1.04 (0.72–1.49)	26	0.81 (0.50–1.32)	18	0.68 (0.38–1.20)	16	1.08 (0.56–2.08)
> 12	3561	25	0.93 (0.58–1.49)	16	0.84 (0.47–1.50)	10	0.65 (0.32–1.33)	10	1.02 (0.45–2.27)
*p*-trend			0.855		0.425		0.139		0.919
*Men—rectum*	25,073	53							
< 8	15,144	31	1.00 (ref.)						
8–12	6368	17	1.43 (0.77–2.64)						
> 12	3561	5	0.70 (0.27–1.82)						
*p*-trend			0.892						
**Occupational sitting time (h/day)**									
*Men—colon*	25,073	190		114		87		58	
> 6	6511	57	1.00 (ref.)	34	1.00 (ref.)	22	1.00 (ref.)	14	1.00 (ref.)
2–6	11,617	87	0.81 (0.56–1.17)	48	0.76 (0.48–1.20)	47	1.15 (0.67–1.96)	27	0.98 (0.49–1.94)
< 2	6944	46	0.75 (0.49–1.14)	32	0.84 (0.51–1.39)	18	0.73 (0.37–1.41)	17	0.99 (0.46–2.13)
*p*-trend			0.181		0.508		0.325		0.992
*Men—rectum*	25,073	53							
> 6	6511	17	1.00 (ref.)						
2–6	11,617	20	0.61 (0.32–1.19)						
< 2	6944	16	0.92 (0.46–1.86)						
*p*-trend			0.826						

*HR* hazard ratio; *CI* confidence interval; *CRC* colorectal cancer; *(d/p)MMR* mismatch repair (deficient/proficient); *NLCS* Netherlands Cohort Study; *p-het* *p*-heterogeneity

*p*-heterogeneity tests (reference group for all tests: wild-type for *KRAS, PIK3CA,* and *BRAF,* and pMMR) were not statistically significant

^a^Hazard ratios were adjusted for age (years; continuous), BMI (kg/m^2^), total energy intake (kcal/day; continuous), family history of CRC (yes/no), alcohol consumption (0; 0.1–4; 5–14; > 15 g/day), processed meat intake (g/day; continuous), red meat intake (g/day; continuous). Age was included as a time-varying covariate

^b^Analyses for subgroups with < 50 cases were not performed

#### Adiposity

BMI and clothing-size were both associated with an increased risk of overall colon cancer in men (Table 3). Associations were similarly positive for the all-wild-type + pMMR subgroup [BMI: HR_5kg/m2_ (95%-CI): 1.34 (1.08–1.67), *p*-trend_quartiles_: 0.038; clothing-size: HR_two sizes_: 1.34 (1.12–1.61), *p*-trend_quartiles_: 0.008] and the any-mutation/dMMR subgroup [BMI: HR_5kg/m2_ (95%-CI): 1.28 (1.07–1.53), *p*-trend_quartiles_: 0.027; clothing-size: HR_two sizes_: 1.32 (1.11–1.55), *p*-trend_quartiles_: 0.002]. Although positive associations were found across all subgroups of individual molecular features (Table 4), associations were strongest for the *PIK3CA*_mut_ subgroup [BMI: HR_5kg/m2_ (95%-CI): 1.38 (1.05–1.82), *p*-trend_quartiles_: 0.007; clothing-size: HR_two sizes_: 1.31 (1.01–1.70), *p*-trend_quartiles_: 0.094], and weakest for the *BRAF*_mut_ subgroup [BMI: HR_5kg/m2_ (95%-CI): 1.23 (0.87–1.72), *p*-trend_quartiles_: 0.603; clothing-size: HR_two sizes_: 1.18 (0.85–1.64), *p*-trend_categories_: 0.360]. In women, BMI and clothing-size were not associated with risk of overall colon cancer, nor with the all-wild-type + pMMR or any-mutation/dMMR subgroups (Table 3). For individual molecular features, both BMI and clothing-size were associated with an increased risk of *KRAS*_mut_ [BMI: HR_5kg/m2_ (95% CI): 1.31 (1.10–1.57), *p*-trend_quartiles_: 0.031; clothing-size: HR_two sizes_: 1.26 (1.03–1.53), *p*-trend_quartiles_: 0.229], but not with *PIK3CA*_mut_, *BRAF*_mut_, or dMMR colon cancer in women (Table 4). No associations between BMI or clothing-size and risk of overall rectal cancer were observed in men or in women, and stratification on subgroups did not lead to clear associations (Tables 3, 4). None of the models with mutual adjustment for BMI and clothing-size showed clear associations of BMI or clothing-size with CRC subgroups based on molecular features (Supplementary Tables S9–S10).

#### Non-occupational physical activity

Non-occupational physical activity was not associated with overall colon cancer risk in men (Table 5). However, a borderline significant inverse association was found between non-occupational physical activity and risk of the any-mutation/dMMR subgroup [HR_30min/day_ (95% CI): 0.97 (0.92–1.02), *p*-trend_categories_: 0.050], whereas no association was found for the all-wild-type + pMMR subgroup. Other subgroups of molecular features in colon cancer did not show clear associations (Table 6). In contrast, non-occupational physical activity was associated with an increased risk of overall rectal cancer in men, which was stronger for the any-mutation/dMMR subgroup [HR_>90vs≤30 min/day_ (95% CI): 3.32 (1.28–8.60), *p*-trend_categories_: 0.033], whereas no clear association was found for the all-wild-type + pMMR or *KRAS*_mut_ subgroups (Tables 5, 6). However, it should be noted that the reference group (≤ 30 min/day) in the any-mutation/dMMR and *KRAS*_mut_ subgroups had a limited number of cases (*n* = 5). In women, non-occupational physical activity was associated with a decreased risk of overall colon cancer (Table 5). Although inverse associations were found for all subgroups, most did not reach statistical significance (Tables 5, 6). Only the any-mutation/dMMR subgroup [HR_>90vs≤30 min/day_ (95% CI): 0.71 (0.51–0.98), *p*-trend_categories_: 0.024] and the subgroup with a *PIK3CA*_mut_-tumor [HR_>90vs≤30 min/day_ (95% CI): 0.51 (0.28–0.93), *p*-trend_categories_: 0.042] showed statistically significant inverse associations. Non-occupational physical activity was not associated with overall rectal cancer in women, and stratification on subgroups did not lead to clear associations (Tables 5, 6).

#### Occupational physical activity

Occupational energy expenditure was associated with a decreased risk of overall colon cancer in men (Table 5). Even though inverse associations were observed for both combination subgroups, only the association with the all-wild-type + pMMR subgroup reached statistical significance [HR_>12 kJ/min_ (95% CI): 0.51 (0.30–0.84), *p*-trend_categories_: 0.006]. Furthermore, lower occupational sitting time was associated with a decreased risk of overall colon cancer in men (Table 5), and associations were slightly stronger for the all-wild-type + pMMR subgroup [HR_<2 h/day_ (95% CI): 0.56 (0.38–0.81), *p*-trend_categories_: 0.003] compared to the any-mutation/dMMR subgroup [HR_<2 h/day_ (95% CI): 0.70 (0.50–0.97), *p*-trend_categories_: 0.034]. No associations were observed for occupational physical activity measures and subgroups of individual molecular features in colon cancer (Table 6). Occupational physical activity measures were not associated with risk of rectal cancer in men, and stratification on subgroups did not lead to clear associations (Tables 5, 6).

#### Heterogeneity testing

For heterogeneity analyses, the all-wild-type + pMMR subgroup served as the reference group for all other subgroups (i.e. any-mutation/dMMR, *KRAS*_mut_, *PIK3CA*_mut_, *BRAF*_mut_, and dMMR). Statistically significant heterogeneity was observed only for BMI associations between *KRAS*_mut_ versus all-wild-type + pMMR colon cancer in women (*p* = 0.008), but not for any other subgroup.

#### Sensitivity analyses

Sensitivity analyses excluding the first two years of follow-up did not lead to essential changes (*data not shown*).

## Discussion

In this large prospective cohort study, we investigated associations between energy balance-related factors and risk of CRC subgroups based on *KRAS*_mut_, *PIK3CA*_mut_, *BRAF*_mut_, and MMR status. Associations between energy balance-related factors and risk of CRC varied by abovementioned molecular features, as well by sex and tumor location. A statistically significant difference in associations was only found between all-wild-type + pMMR and *KRAS*_mut_ subgroups of colon cancer in women regarding BMI associations. In women, we observed positive associations for BMI and clothing-size with risk of *KRAS*_mut_ colon cancer, but not with any other subgroup. In men, BMI and clothing-size were positively associated with risk of colon, but not rectal cancer, regardless of molecular features subgroups. While positive associations of BMI and clothing-size with risk of colon cancer were observed in men for all individual molecular features, associations were strongest for *PIK3CA*_mut_ tumors and weakest for *BRAF*_mut_ tumors. Non-occupational physical activity was inversely associated with any-mutation/dMMR colon cancer in men and women, but not with all-wild-type + pMMR colon cancer. In men, no clear associations were observed between non-occupational physical activity and individual molecular features in colon cancer. In women, inverse associations were observed for all individual molecular features, but associations were strongest for *PIK3CA*_mut_ colon cancer. Occupational physical activity was associated with a decreased risk of colon cancer for both combination subgroups in men, but associations were strongest for all-wild-type + pMMR tumors.

Several studies have focused on investigating associations between energy balance-related factors (i.e. BMI, waist-circumference, physical activity) and risk of CRC in relation to specific (individual) mutations and/or MSI/MMR status, but results have been inconsistent (Carr et al. 2018, 2020; Myte et al. 2019; Brändstedt et al. 2014; Slattery et al. 2000, 2001; Hughes et al. 2012; Campbell et al. 2010; Hoffmeister et al. 2013). To our knowledge, the current study is the first to combine cases into subgroups based on *KRAS*_mut_, *PIK3CA*_mut_, *BRAF*_mut_, and MMR status, and study potential etiological differences between these subgroups. Instead of comparing wild-type versus mutated tumors for individual genes and proficient versus deficient tumors for MMR, as done in previous studies, the all-wild-type + pMMR subgroup served as the reference group for all other subgroups in the current study. Combining mutation and MMR status into subgroups has some advantages. First, it has been suggested that mutations in *KRAS*, *PIK3CA*, and *BRAF* drive metabolic reprogramming toward the Warburg-effect (Levine and Puzio-Kuter 2010; Kimmelman 2015; Hutton et al. 2016; Jiang et al. 2018), and we have shown previously that MMR deficiency is associated with presence of the Warburg-effect (Offermans et al. 2021). Combining these molecular features, presumed to be involved in the same metabolic phenotype, thus results in a cleaner reference group compared to groups based on individual features (e.g. *KRAS* mutated versus wild-type). Our results show that co-occurrence of *KRAS*_mut_ and *PIK3CA*_mut_ is relatively common, as is co-occurrence of *BRAF*_mut_ and dMMR. Using the all-wild-type + pMMR subgroup as the reference for all subgroups of individual mutations and MMR status, this reference group is less heterogeneous compared to, e.g., the *KRAS* wild-type (*KRAS*_wt_) group, which still contains a large number of cases with a *PIK3CA* mutation. Second, differentiating subgroups on the basis of the combination of presence or absence of mutations and/or dMMR leads to increased statistical power, since most individual molecular features occurred in < 20% of CRC cases (e.g., MMR deficiency: 10.7%).

Previous studies on adiposity and risk of CRC in relation to molecular features mainly focused on BMI (Carr et al. 2018, 2020; Myte et al. 2019; Brändstedt et al. 2013, 2014; Slattery et al. 2000, 2001, 2007; Hughes et al. 2012; Campbell et al. 2010; Hoffmeister et al. 2013; Hanyuda et al. 2016), though some used additional adiposity measures like waist circumference (Brändstedt et al. 2013, 2014; Hughes et al. 2012). Two cohort studies (Myte et al. 2019; Brändstedt et al. 2014) and two case–control studies (Carr et al. 2020; Slattery et al. 2001) investigated adiposity in relation to *KRAS*_mut_ status in CRC. Our results are in line with those of Slattery et al. (2001), which showed positive associations of adiposity with *KRAS*_mut_ but not *KRAS*_wt_ colon cancer in women, whereas similar associations were observed for *KRAS*_mut_ and *KRAS*_wt_ in men. A study by Brändstedt et al. (2014) also reported positive associations between adiposity and *KRAS*_mut_ but not *KRAS*_wt_ CRC, but in men, not women. These and our results are in contrast with those of Carr et al. (2020) and Myte et al. (2019), who reported positive associations of adiposity with *KRAS*_wt_ CRC (note: *KRAS*_wt_ + *BRAF*_wt_ in the study by Myte et al.) but no or weak associations with *KRAS*_mut_ CRC. Three cohort studies (Myte et al. 2019; Brändstedt et al. 2014; Hughes et al. 2012), including one study that used data from the NLCS with 7.3 years of follow-up (Hughes et al. 2012), and two case–control studies (Carr et al. 2020; Slattery et al. 2007) studied adiposity in relation to *BRAF*_mut_ status in CRC. Our results are in line with all but one of these studies (Myte et al. 2019; Brändstedt et al. 2014; Hughes et al. 2012; Slattery et al. 2007), as these reported either a weaker positive association of adiposity with *BRAF*_mut_ compared to *BRAF*_wt_ CRC (Brändstedt et al. 2014; Hughes et al. 2012), or no association with *BRAF*_mut_ CRC (Myte et al. 2019; Slattery et al. 2007). Even though Carr et al. (2020) observed this same difference in associations for men, associations between adiposity and CRC were stronger for *BRAF*_mut_ CRC than *BRAF*_wt_ CRC in women. For MSI/MMR status, our results are in line with those of a recent meta-analysis by Carr et al. (2018), in which no difference in associations was observed between adiposity and MSI status in CRC. Our study is the first to investigate the association between adiposity and CRC risk in relation to *PIK3CA*_mut_ status, and therefore cannot be compared to any previous data.

To our knowledge, associations between physical activity and colon cancer risk in relation to molecular features have only been investigated in a case–control study by Slattery et al. for *KRAS*_mut_ (Slattery et al. 2001), *BRAF*_mut_ (Slattery et al. 2007), and MSI (Slattery et al. 2000) status. Our results are partly in line with these studies, which showed stronger positive associations between physical inactivity and risk of *KRAS*_mut_ colon cancer compared to *KRAS*_wt_ colon cancer in men, whereas associations did not differ according to *KRAS*_mut_ status in women (Slattery et al. 2001). For *BRAF*, they observed no association between physical activity and *BRAF*_mut_ colon cancer (Slattery et al. 2007). Lastly, physical activity was associated with both MSS and MSI colon cancer in men, but only with MSS colon cancer in women (Slattery et al. 2000). Our results for *PIK3CA*_mut_ CRC cannot be compared to any previous data, since studies investigating associations between physical activity and *PIK3CA*_mut_ status in CRC are currently lacking.

The contradicting results across molecular pathological epidemiology (MPE) studies regarding associations of energy balance-related factors with risk of CRC according to *KRAS*_mut_, *BRAF*_mut_, and/or MSI/MMR status might be attributed to several factors. For example: use of different methods for assessing molecular features (e.g. assessment of different mutations or MSI versus MMR status); different timing and method of exposure measurements (i.e. BMI, waist circumference, physical activity); different study designs (i.e. cohort versus case–control); different approaches for (outcome) stratification (for example stratification on sex and tumor location); and/or chance findings due to multiple testing, caused by repeatedly splitting CRC into different molecular pathological subgroups. We therefore believe it is important that large prospective cohort studies replicate the current analyses, preferably stratified on tumor location and sex.

The current results suggest a role of *KRAS* mutations in the etiological pathway between adiposity and colon cancer risk in women (adiposity was only associated with *KRAS*_mut_ colon cancers). In contrast, our results do not indicate a clear role of one of the molecular features in the etiological pathway between adiposity and colon cancer in men (adiposity was associated with all subgroups of molecular features in colon cancer). As mentioned above, the molecular features used in the current study have all been associated with the Warburg-effect (Levine and Puzio-Kuter 2010; Kimmelman 2015; Hutton et al. 2016; Jiang et al. 2018; Offermans et al. 2021). Associations with the all-wild-type + pMMR group indicate a low likelihood of Warburg-effect involvement, whereas associations with the any-mutation/dMMR subgroup or subgroups of individual molecular features indicate a higher likelihood of Warburg-effect involvement. Therefore, the current results indicate a potential role of the Warburg-effect in the etiological pathway between adiposity and colon cancer in women through *KRAS* mutations, but not other molecular features. In men, a role of the Warburg-effect in the etiological pathway between adiposity and colon cancer is not indicated by the current results. In a previous study, we investigated associations between energy balance-related factors and risk of Warburg-subtypes in CRC, based IHC expression of proteins involved in the Warburg-effect (Jenniskens et al. 2021a). The results of this previous study indicated involvement of the Warburg-effect in associations between adiposity and colon cancer risk in both men and women, though additional mechanisms could be at play in women as well.

For physical activity, the current results indicate a role of molecular features (*KRAS*_mut_, *PIK3CA*_mut_, *BRAF*_mut_, and/or MMR deficiency) in the etiological pathway between physical inactivity and colon cancer risk in women (physical activity was associated with any-mutation/dMMR colon cancer), and it seems that in particular *PIK3CA* mutations are involved in this association (strongest association observed with *PIK3CA*_mut_ colon cancer). In men, the current results do not give a clear indication of involvement of molecular features in the association between physical activity and colon cancer. While non-occupational physical activity was inversely associated with the any-mutation/dMMR subgroup, occupational physical activity was mainly associated with the all-wild-type + pMMR subgroup. It is assumed that occupational physical activity gives a better indication of physical activity for men than non-occupational physical activity. That is, while occupational physical activity represents long-term physical activity (median duration of longest held job: 29 years), non-occupational physical activity probably reflects the last few years before baseline. Therefore, the current results suggest that the molecular features studied here are not involved in the etiological pathway between physical inactivity and colon cancer risk in men. All in all, the current results indicate involvement of the Warburg-effect in associations between physical activity and colon cancer risk in women, but not men. Results of our previous study on Warburg-subtypes in CRC indicated that inverse associations between physical activity and colon cancer risk are explained by mechanisms other than the Warburg-effect (Jenniskens et al. 2021a).

Altogether, results from our previous study on Warburg-subtypes in CRC are only partly in line with the current results. Although the molecular features that were considered in the current study have been associated with the Warburg-effect (Levine and Puzio-Kuter 2010; Kimmelman 2015; Hutton et al. 2016; Jiang et al. 2018; Offermans et al. 2021), they are additionally known for their involvement in numerous diverse (oncogenic) cellular pathways for cell growth, differentiation, proliferation, and survival (Li et al. 2020; Haluska et al. 2007; Boland and Goel 2010). Therefore, the molecular features used in the current study might not always be a good reflection of the Warburg-effect. Furthermore, tumors of cases in the all-wild-type + pMMR subgroup might express other molecular features, possibly also associated with the Warburg-effect, that were not assessed in the current study. This may have potentially influenced our results. Still, combining these molecular features into all-wild-type + pMMR and any-mutation/dMMR subgroups seemed to be a straightforward way of subgrouping CRC cases, especially for physical activity associations.

A major strength of the current study is the prospective cohort design with long follow-up (20.3 years) and availability of DNA from FFPE tumor material from a large number of incident CRC cases. Another strength was the detection of mutations using MassARRAY technology, which has been shown to be a suitable technique for mutation typing in (older) FFPE material (Fleitas et al. 2016). The ColoCarta panel that was used includes assays for most of the *KRAS* (99%) and *BRAF* (98%) mutations, but it identifies only 78% of known *PIK3CA* mutations (Fumagalli et al. 2010). However, the most common *PIK3CA* mutations are included (Gray et al. 2017). This makes it unlikely that additional detection of less common mutations would alter the current results, since the number of additional cases with a *PIK3CA* mutation would be rather small. As an indicator of MSI status, we used IHC expression of MLH1 and MSH2, which might have led to misclassification of some of the cases. However, it has been shown that loss of MLH1 or MSH2 expression was observed in ~ 90% of MSI cases (Lanza et al. 2002).

In conclusion, results from this large prospective cohort study provide further insights in the associations between energy balance-related factors and CRC risk according to *KRAS*_mut_, *PIK3CA*_mut_, *BRAF*_mut_, and MMR status. Associations between energy balance-related factors and risk of CRC varied by these molecular features, as well by sex and tumor location. Our results suggest a role of *KRAS* mutations in the etiological pathway between adiposity and colon cancer in women. For men, our results do not indicate a role of one of the molecular features in the etiological pathway of adiposity and colon cancer. Furthermore, the current results indicate a role of mutations in *KRAS*, *PIK3CA*, and/or *BRAF*, and/or MMR deficiency in the etiological pathway between physical inactivity and colon cancer risk in women, but not men, and it seems that in particular *PIK3CA* mutations are involved in this association. Our findings need to be replicated in additional large-scale MPE-studies.

## Supplementary Information

Below is the link to the electronic supplementary material.

Supplementary file1 (DOCX 132 KB)
