# Supplementary material for: Energy balance-related factors and risk of colorectal cancer based on KRAS, PIK3CA, and BRAF mutations and MMR status
Source: J Cancer Res Clin Oncol. 2022 May 11;148(10):2723–42. doi: 10.1007/s00432-022-04019-9 (PMC9470639; doi:10.1007/s00432-022-04019-9)
Supplement: Supplementary file 1 — Supplementary file1 (DOCX 132 KB) [file 432_2022_4019_MOESM1_ESM.docx]

# Supplementary Materials for

**Energy balance-related factors and risk of colorectal cancer based on KRAS, PIK3CA, and BRAF mutations and MMR status**

Josien C. A. Jenniskens, Kelly Offermans, Colinda C. J. M. Simons, Iryna Samarska, Gregorio E. Fazzi, Jaleesa R. M. van der Meer, Kim M. Smits, Leo J. Schouten, Matty P. Weijenberg, Heike I. Grabsch,

Piet A. van den Brandt

This PDF file includes:

Supplementary Methods

Supplementary Table S1-S10

# Supplementary Methods

## Deparaffinization of FFPE sections

FFPE tissue sections containing tumor primary tumor were deparaffinized using an adapted version of the protocol for Purification of genomic DNA from FFPE tissue using the QIAamp® DNA FFPE Tissue Kit and Deparaffinization Solution (Qiagen, Hilden, Germany). The adapted protocol included the following steps: I) Add 320 μl Deparaffinization Solution to 2 x 20 µm sections and vortex vigorously for 10 s. Centrifuge briefly to collect the sample in the bottom of the tube; II) Incubate at 56°C for 3 min, and then allow to cool at room temperature (15–25°C); III) Add 200 μl Buffer ATL, and mix by vortexing; IV) Centrifuge for 1 min at 11,000 x g (10,000 rpm); V) Add 20 μl proteinase K to the lower clear phase and mix gently by pipetting up and down; VI) Incubate at 56°C for 1 h; VII) Mix clear phase by pipetting up and down; VIII) Incubate at 90°C for 1 h; IX) Briefly centrifuge the 1.5 ml tube to remove drops from inside the lid; X) Transfer the 2 ml microcentrifuge tubes to QiaSymphony.

| **Supplementary Table S1 –** ColoCarta panel genes and mutations | | |
| --- | --- | --- |
| **Gene** | **Assay** | **Mutation** |
|  |  |  |
| *BRAF* | 15/16 | *V600E/K/L/M/R* |
|  | 9 | *D594G/V* |
|  |  |  |
| *KRAS* | 1 | *G12A/D/V* |
|  | 2 | *G12C/R/S* |
|  | 4 | *G13D/V* |
|  | 5 | *A59T* |
|  | 7 | *Q61L/P/R* |
|  | 8 | *Q61H_A/H_G* |
|  |  |  |
| *PIK3CA* | 1 | *R88Q* |
|  | 3 | *C420R* |
|  | 5 | *E542K* |
|  | 6 | *E545K* |
|  | 7 | *Q546K* |
|  | 8 | *H701P* |
|  | 9 | *H1047L/R* |
|  |  |  |
| *NRAS* | 1 | *G12A/D/V* |
|  | 2 | *G12C/R/S* |
|  | 3 | *G13A/D/V* |
|  | 4 | *G13C/R/S* |
|  | 7 | *Q61H* |
|  | 8 | *Q61E/K* |
|  |  |  |
| *HRAS* | 6 | *Q61L/P/R* |
|  |  |  |
| *MET* | 1 | *R970C* |
|  | 2 | *T992I* |
|  |  |  |

| **Supplementary Table S2 –** Baseline characteristics [mean (SD) or %] of CRC cases by availability of mutation and MMR status; NLCS, 1986-2006. | | |
| --- | --- | --- |
|  | **Information on mutation and MMR status** | |
|  | **Not available** | **Available** |
|  |  |  |
| N | 2248 | 2349 |
| Men (%) | 55.7 | 56.1 |
| Age | 62.1 (4.2) | 61.8 (4.1) |
| Overweight/obesity^a^ (%) | 49.0 | 50.0 |
| Clothing size^b^ | 48.1 (5.0) | 48.2 (5.1) |
| Non-occupational physical activity >60 min/day | 47.5 | 46.6 |
| Occupational energy expenditure >12 kJ/min^c^ | 11.2 | 10.7 |
| Occupational sitting time <2 hours/day^c^ | 33.1 | 32.7 |
| Height (cm) | 171.8 (8.4) | 172.1 (8.5) |
| Total energy intake (kcal/day) | 1936 (504) | 1944 (492) |
| Family history of colorectal cancer (%) | 8.4 | 9.5 |
| Alcohol consumption (g/day) | 12.2 (16.2) | 11.4 (14.8) |
| Processed meat intake (g/day) | 14.0 (15.7) | 13.2 (14.2) |
| Red meat intake (g/day) | 87.7 (41.2) | 87.5 (39.6) |
| Never cigarette smokers (%) | 32.9 | 32.1 |
| University or higher vocational education (%) | 14.3 | 15.1 |
|  |  |  |
| Abbreviations: SD, standard deviation; CRC, colorectal cancer; MMR, mismatch repair; NLCS, Netherlands Cohort Study.  ^a^Body mass index ≥25.  ^b^Lower body clothing size. Based on fewer participants due to extra missings.  ^c^Based on fewer participants due to shorter follow-up (17.3 years), only available for men. | | |

| **Supplementary Table S3 -** Age-adjusted HRs^a^ and 95%-CIs for associations between adiposity measures and CRC in subgroups based on mutation and MMR status, by sex and tumor location; NLCS, 1986-2006. | | | | | | | | | | | | | | |  |
| --- | --- | --- | --- | --- | --- | --- | --- | --- | --- | --- | --- | --- | --- | --- | --- |
|  | **Person-years at risk** | **Total** | | | | **Wild-type+pMMR^b^** | | | | | **Any-mutation/dMMR^c^** | | | | |
|  |  | n_cases_ | HR (95%-CI) | | | n_cases_ | HR (95%-CI) | | | | n_cases_ | HR (95%-CI) | | | |
| **BMI quartiles (kg/m^2^): range (median)** | | | |  |  | | |  |  | | | |  |  |  |
|  |  |  |  | | |  |  | | | |  |  | | | |
| **Men – colon** |  |  |  | | |  |  | | | |  |  | | | |
| < 23.4 (22.2) | 7993 | 167 | 1.00 (ref.) | | | 66 | 1.00 (ref.) | | | | 101 | 1.00 (ref.) | | | |
| 23.4-24.9 (24.2) | 8343 | 188 | 1.06 (0.83-1.36) | | | 79 | 1.13 (0.79-1.60) | | | | 109 | 1.02 (0.75-1.38) | | | |
| 25.0-26.6 (25.7) | 7683 | 200 | 1.23 (0.96-1.57) | | | 77 | 1.20 (0.84-1.72) | | | | 123 | 1.24 (0.92-1.67) | | | |
| > 26.6 (27.8) | 7003 | 199 | 1.35 (1.06-1.74) | | | 87 | 1.51 (1.06-2.14) | | | | 112 | 1.25 (0.92-1.70) | | | |
| P-trend |  |  | 0.008 | | |  | 0.021 | | | |  | 0.069 | | | |
| per 5 kg/m^2^ | 31022 | 754 | 1.28 (1.11-1.48) | | | 309 | 1.35 (1.09-1.67) | | | | 445 | 1.23 (1.04-1.46) | | | |
| **Men – rectum** |  |  |  | | |  |  | | | |  |  | | | |
| < 23.4 (22.2) | 7993 | 58 | 1.00 (ref.) | | | 34 | 1.00 (ref.) | | | | 24 | 1.00 (ref.) | | | |
| 23.4-24.9 (24.2) | 8343 | 54 | 0.88 (0.59-1.30) | | | 35 | 0.97 (0.60-1.59) | | | | 19 | 0.75 (0.40-1.38) | | | |
| 25.0-26.6 (25.7) | 7683 | 65 | 1.16 (0.80-1.70) | | | 42 | 1.29 (0.80-2.07) | | | | 23 | 0.99 (0.55-1.77) | | | |
| > 26.6 (27.8) | 7003 | 47 | 0.93 (0.62-1.40) | | | 24 | 0.81 (0.47-1.40) | | | | 23 | 1.08 (0.60-1.95) | | | |
| P-trend |  |  | 0.881 | | |  | 0.824 | | | |  | 0.622 | | | |
| per 5 kg/m^2^ | 31022 | 224 | 1.02 (0.82-1.27) | | | 135 | 0.95 (0.72-1.25) | | | | 89 | 1.13 (0.81-1.58) | | | |
| **Women – colon** |  |  |  | | |  |  | | | |  |  | | | |
| <22.8 (21.5) | 9014 | 181 | 1.00 (ref.) | | | 56 | 1.00 (ref.) | | | | 125 | 1.00 (ref.) | | | |
| 22.8-24.7 (23.8) | 8914 | 146 | 0.80 (0.62-1.03) | | | 43 | 0.77 (0.51-1.17) | | | | 103 | 0.81 (0.61-1.09) | | | |
| 24.8-27.0 (25.7) | 8141 | 147 | 0.89 (0.69-1.15) | | | 36 | 0.71 (0.46-1.10) | | | | 111 | 0.97 (0.73-1.30) | | | |
| >27.0 (29.2) | 8158 | 156 | 0.94 (0.73-1.20) | | | 44 | 0.87 (0.57-1.31) | | | | 112 | 0.97 (0.73-1.29) | | | |
| P-trend |  |  | 0.770 | | |  | 0.432 | | | |  | 0.890 | | | |
| per 5 kg/m^2^ | 34228 | 630 | 1.02 (0.90-1.15) | | | 179 | 0.87 (0.69-1.09) | | | | 451 | 1.08 (0.95-1.24) | | | |
| **Women – rectum** |  |  |  | | |  |  | | | |  |  | | | |
| <22.8 (21.5) | 9014 | 37 | 1.00 (ref.) | | | 16 | 1.00 (ref.) | | | | 21 | 1.00 (ref.) | | | |
| 22.8-24.7 (23.8) | 8914 | 26 | 0.71 (0.42-1.19) | | | 15 | 0.96 (0.47-1.95) | | | | 11 | 0.52 (0.25-1.10) | | | |
| 24.8-27.0 (25.7) | 8141 | 32 | 0.96 (0.59-1.57) | | | 18 | 1.26 (0.64-2.49) | | | | 14 | 0.73 (0.37-1.46) | | | |
| >27.0 (29.2) | 8158 | 36 | 1.07 (0.67-1.73) | | | 15 | 1.05 (0.52-2.14) | | | | 21 | 1.09 (0.59-2.03) | | | |
| P-trend |  |  | 0.565 | | |  | 0.696 | | | |  | 0.661 | | | |
| per 5 kg/m^2^ | 34228 | 131 | 1.10 (0.89-1.36) | | | 64 | 1.08 (0.80-1.46) | | | | 67 | 1.12 (0.84-1.49) | | | |
|  |  |  |  | | |  |  | | | |  |  | | | |
| **Clothing size: range (median)** | | | |  |  | | |  |  | | | |  |  |  |
|  |  |  |  | | |  |  | | |  | |  | | | |
| **Men – colon** |  |  |  | | |  |  | | |  | |  | | | |
| ≤50 (50) | 10903 | 211 | 1.00 (ref.) | | | 90 | 1.00 (ref.) | | | 121 | | 1.00 (ref.) | | | |
| 52 (52) | 9750 | 247 | 1.27 (1.02-1.59) | | | 104 | 1.27 (0.94-1.73) | | | 143 | | 1.28 (0.97-1.67) | | | |
| 54 (54) | 5156 | 136 | 1.33 (1.02-1.72) | | | 53 | 1.23 (0.85-1.78) | | | 83 | | 1.40 (1.02-1.92) | | | |
| ≥56 (56) | 2619 | 90 | 1.76 (1.29-2.39) | | | 39 | 1.82 (1.20-2.77) | | | 51 | | 1.71 (1.18-2.50) | | | |
| P-trend |  |  | <0.001 | | |  | 0.011 | | |  | | 0.002 | | | |
| per 2 sizes | 28428 | 684 | 1.31 (1.15-1.49) | | | 286 | 1.32 (1.11-1.58) | | | 398 | | 1.30 (1.11-1.53) | | | |
| **Men – rectum** |  |  |  | | |  |  | | |  | |  | | | |
| ≤50 (50) | 10903 | 78 | 1.00 (ref.) | | | 46 | 1.00 (ref.) | | | 32 | | 1.00 (ref.) | | | |
| 52 (52) | 9750 | 69 | 0.99 (0.70-1.40) | | | 46 | 1.14 (0.74-1.74) | | | 23 | | 0.79 (0.46-1.36) | | | |
| 54 (54) | 5156 | 43 | 1.18 (0.79-1.76) | | | 25 | 1.18 (0.71-1.97) | | | 18 | | 1.17 (0.65-2.11) | | | |
| ≥56 (56) | 2619 | 16 | 0.88 (0.50-1.55) | | | 7 | 0.67 (0.30-1.51) | | | 9 | | 1.16 (0.54-2.47) | | | |
| P-trend |  |  | 0.881 | | |  | 0.782 | | |  | | 0.580 | | | |
| per 2 sizes | 28428 | 206 | 0.97 (0.80-1.19) | | | 124 | 0.96 (0.75-1.22) | | | 82 | | 1.00 (0.73-1.37) | | | |
| **Women – colon** |  |  |  | | |  |  | | |  | |  | | | |
| ≤40 (40) | 6574 | 128 | 1.00 (ref.) | | | 46 | 1.00 (ref.) | | | 82 | | 1.00 (ref.) | | | |
| 42 (42) | 8582 | 150 | 0.88 (0.67-1.16) | | | 34 | 0.57 (0.36-0.91) | | | 116 | | 1.06 (0.77-1.45) | | | |
| 44 (44) | 9270 | 159 | 0.84 (0.64-1.11) | | | 48 | 0.74 (0.48-1.13) | | | 111 | | 0.90 (0.65-1.24) | | | |
| ≥46 (46) | 9454 | 182 | 0.93 (0.71-1.22) | | | 50 | 0.76 (0.50-1.16) | | | 132 | | 1.04 (0.76-1.42) | | | |
| P-trend |  |  | 0.664 | | |  | 0.481 | | |  | | 0.938 | | | |
| per 2 sizes | 33880 | 619 | 1.08 (0.94-1.23) | | | 178 | 1.06 (0.82-1.39) | | | 441 | | 1.08 (0.94-1.24) | | | |
| **Women – rectum** |  |  |  | | |  |  | | |  | |  | | | |
| ≤40 (40) | 6574 | 23 | 1.00 (ref.) | | | 11 | 1.00 (ref.) | | | 12 | | 1.00 (ref.) | | | |
| 42 (42) | 8582 | 30 | 0.99 (0.57-1.74) | | | 17 | 1.20 (0.55-2.59) | | | 13 | | 0.81 (0.37-1.80) | | | |
| 44 (44) | 9270 | 35 | 1.06 (0.62-1.84) | | | 20 | 1.31 (0.62-2.76) | | | 15 | | 0.85 (0.39-1.86) | | | |
| ≥46 (46) | 9454 | 42 | 1.25 (0.74-2.13) | | | 16 | 1.04 (0.48-2.28) | | | 26 | | 1.43 (0.70-2.89) | | | |
| P-trend |  |  | 0.348 | | |  | 0.920 | | |  | | 0.239 | | | |
| per 2 sizes | 33880 | 130 | 1.02 (0.83-1.26) | | | 64 | 0.98 (0.72-1.32) | | | 66 | | 1.07 (0.81-1.43) | | | |
|  |  |  |  | | |  |  | | |  | |  | | | |
| Abbreviations: HR, hazard ratio; CI, confidence interval; CRC, colorectal cancer; (d/p)MMR, mismatch repair (deficient/proficient); NLCS, Netherlands Cohort Study; BMI, body mass index.  ^a^Hazard Ratios were adjusted for age (years; continuous) and age was included as a time-varying covariate.  ^b^This group excludes cases with mutations in any of the genes (*KRAS*, *BRAF*, or *PIK3CA*), as well as MMR deficient cases.  ^c^This group includes cases with mutations in any of the genes (*KRAS*, *BRAF*, or *PIK3CA*) and/or cases that are MMR deficient. | | | | | | | | | | | | | | |  |

| **Supplementary Table S4 -** Age-adjusted HRs^a^ and 95%-CIs for associations between adiposity measures and CRC for individual mutations and MMR status, by sex and tumor location; NLCS, 1986-2006. | | | | | | | | | | |  |
| --- | --- | --- | --- | --- | --- | --- | --- | --- | --- | --- | --- |
|  | **Person-years at risk** | ***KRAS*_mut_** | | ***PIK3CA*_mut_^b^** | | ***BRAF*_mut_^b^** | | **dMMR^b^** | |  |  |
|  |  | n_cases_ | HR (95%-CI) | n_cases_ | HR (95%-CI) | n_cases_ | HR (95%-CI) | n_cases_ | HR (95%-CI) |  |  |
| **BMI quartiles (kg/m^2^): range (median)** | | | |  |  |  |  |  |  |  |  |
|  |  |  |  |  |  |  |  |  |  |  |  |
| **Men – colon** | |  |  |  |  |  |  |  |  |  |  |
| < 23.4 (22.2) | 7993 | 61 | 1.00 (ref.) | 27 | 1.00 (ref.) | 25 | 1.00 (ref.) | 19 | 1.00 (ref.) |  |  |
| 23.4-24.9 (24.2) | 8343 | 58 | 0.90 (0.61-1.31) | 38 | 1.33 (0.80-2.22) | 28 | 1.05 (0.60-1.84) | 13 | 0.65 (0.32-1.33) |  |  |
| 25.0-26.6 (25.7) | 7683 | 69 | 1.15 (0.80-1.67) | 42 | 1.60 (0.97-2.64) | 29 | 1.18 (0.68-2.05) | 21 | 1.11 (0.59-2.10) |  |  |
| > 26.6 (27.8) | 7003 | 68 | 1.26 (0.87-1.83) | 43 | 1.81 (1.09-2.99) | 23 | 1.03 (0.57-1.84) | 17 | 1.00 (0.51-1.95) |  |  |
| P-trend |  |  | 0.122 |  | 0.013 |  | 0.808 |  | 0.659 |  |  |
| per 5 kg/m^2^ | 31022 | 256 | 1.24 (1.00-1.53) | 150 | 1.33 (1.03-1.72) | 105 | 1.18 (0.86-1.63) | 70 | 1.38 (0.92-2.07) |  |  |
| **Men – rectum** | |  |  |  |  |  |  |  |  |  |  |
| < 23.4 (22.2) | 7993 | 18 | 1.00 (ref.) |  |  |  |  |  |  |  |  |
| 23.4-24.9 (24.2) | 8343 | 13 | 0.68 (0.33-1.40) |  |  |  |  |  |  |  |  |
| 25.0-26.6 (25.7) | 7683 | 19 | 1.08 (0.56-2.08) |  |  |  |  |  |  |  |  |
| > 26.6 (27.8) | 7003 | 18 | 1.12 (0.58-2.19) |  |  |  |  |  |  |  |  |
| P-trend |  |  | 0.494 |  |  |  |  |  |  |  |  |
| per 5 kg/m^2^ | 31022 | 68 | 1.14 (0.79-1.64) |  |  |  |  |  |  |  |  |
| **Women – colon** | |  |  |  |  |  |  |  |  |  |  |
| <22.8 (21.5) | 9014 | 52 | 1.00 (ref.) | 30 | 1.00 (ref.) | 53 | 1.00 (ref.) | 43 | 1.00 (ref.) |  |  |
| 22.8-24.7 (23.8) | 8914 | 46 | 0.88 (0.58-1.33) | 27 | 0.89 (0.52-1.53) | 40 | 0.74 (0.48-1.13) | 34 | 0.77 (0.48-1.24) |  |  |
| 24.8-27.0 (25.7) | 8141 | 65 | 1.37 (0.93-2.02) | 30 | 1.10 (0.65-1.85) | 39 | 0.80 (0.52-1.24) | 24 | 0.61 (0.36-1.02) |  |  |
| >27.0 (29.2) | 8158 | 59 | 1.23 (0.83-1.83) | 29 | 1.05 (0.62-1.78) | 41 | 0.83 (0.54-1.28) | 30 | 0.75 (0.46-1.22) |  |  |
| P-trend |  |  | 0.094 |  | 0.688 |  | 0.469 |  | 0.168 |  |  |
| per 5 kg/m^2^ | 34228 | 222 | 1.27 (1.07-1.51) | 116 | 1.13 (0.88-1.47) | 173 | 0.98 (0.80-1.19) | 131 | 0.89 (0.70-1.14) |  |  |
| **Women – rectum** | |  |  |  |  |  |  |  |  |  |  |
| <22.8 (21.5) | 9014 | 18 | 1.00 (ref.) |  |  |  |  |  |  |  |  |
| 22.8-24.7 (23.8) | 8914 | 7 | 0.39 (0.16-0.94) |  |  |  |  |  |  |  |  |
| 24.8-27.0 (25.7) | 8141 | 9 | 0.55 (0.24-1.24) |  |  |  |  |  |  |  |  |
| >27.0 (29.2) | 8158 | 21 | 1.28 (0.67-2.43) |  |  |  |  |  |  |  |  |
| P-trend |  |  | 0.410 |  |  |  |  |  |  |  |  |
| per 5 kg/m^2^ | 34228 | 55 | 1.20 (0.89-1.63) |  |  |  |  |  |  |  |  |
|  |  |  |  |  |  |  |  |  |  |  |  |
| **Clothing size: range (median)** | | | |  |  |  |  |  |  |  |  |
|  |  |  |  |  |  |  |  |  |  |  |  |
| **Men – colon** | |  |  |  |  |  |  |  |  |  |  |
| ≤50 (50) | 10903 | 73 | 1.00 (ref.) | 40 | 1.00 (ref.) | 30 | 1.00 (ref.) | 18 | 1.00 (ref.) |  |  |
| 52 (52) | 9750 | 84 | 1.23 (0.88-1.73) | 52 | 1.43 (0.93-2.20) | 29 | 1.04 (0.61-1.76) | 23 | 1.35 (0.71-2.55) |  |  |
| 54 (54) | 5156 | 48 | 1.33 (0.89-1.97) | 22 | 1.15 (0.67-1.98) | 21 | 1.40 (0.78-2.49) | 12 | 1.30 (0.61-2.75) |  |  |
| ≥56 (56) | 2619 | 29 | 1.60 (1.00-2.57) | 17 | 1.77 (0.97-3.23) | 9 | 1.20 (0.56-2.60) | 7 | 1.51 (0.62-3.70) |  |  |
| P-trend |  |  | 0.036 |  | 0.117 |  | 0.336 |  | 0.330 |  |  |
| per 2 sizes | 28428 | 234 | 1.23 (1.01-1.51) | 131 | 1.29 (1.00-1.67) | 89 | 1.18 (0.87-1.62) | 60 | 1.31 (0.89-1.93) |  |  |
| **Men – rectum** | |  |  |  |  |  |  |  |  |  |  |
| ≤50 (50) | 10903 | 21 | 1.00 (ref.) |  |  |  |  |  |  |  |  |
| 52 (52) | 9750 | 17 | 0.87 (0.45-1.68) |  |  |  |  |  |  |  |  |
| 54 (54) | 5156 | 17 | 1.65 (0.86-3.18) |  |  |  |  |  |  |  |  |
| ≥56 (56) | 2619 | 9 | 1.74 (0.78-3.86) |  |  |  |  |  |  |  |  |
| P-trend |  |  | 0.077 |  |  |  |  |  |  |  |  |
| per 2 sizes | 28428 | 64 | 1.17 (0.80-1.69) |  |  |  |  |  |  |  |  |
| **Women – colon** | |  |  |  |  |  |  |  |  |  |  |
| ≤40 (40) | 6574 | 35 | 1.00 (ref.) | 24 | 1.00 (ref.) | 29 | 1.00 (ref.) | 32 | 1.00 (ref.) |  |  |
| 42 (42) | 8582 | 54 | 1.16 (0.74-1.81) | 25 | 0.78 (0.44-1.39) | 47 | 1.20 (0.74-1.96) | 35 | 0.82 (0.49-1.35) |  |  |
| 44 (44) | 9270 | 58 | 1.11 (0.71-1.74) | 33 | 0.93 (0.54-1.59) | 45 | 1.02 (0.62-1.66) | 25 | 0.52 (0.30-0.89) |  |  |
| ≥46 (46) | 9454 | 70 | 1.30 (0.84-2.01) | 30 | 0.81 (0.47-1.42) | 49 | 1.07 (0.66-1.73) | 37 | 0.74 (0.45-1.22) |  |  |
| P-trend |  |  | 0.275 |  | 0.650 |  | 0.935 |  | 0.146 |  |  |
| per 2 sizes | 33880 | 217 | 1.24 (1.02-1.50) | 112 | 1.10 (0.86-1.41) | 170 | 1.03 (0.85-1.26) | 129 | 0.90 (0.71-1.13) |  |  |
| **Women – rectum** | |  |  |  |  |  |  |  |  |  |  |
| ≤40 (40) | 6574 | 10 | 1.00 (ref.) |  |  |  |  |  |  |  |  |
| 42 (42) | 8582 | 9 | 0.68 (0.28-1.70) |  |  |  |  |  |  |  |  |
| 44 (44) | 9270 | 12 | 0.83 (0.35-1.97) |  |  |  |  |  |  |  |  |
| ≥46 (46) | 9454 | 23 | 1.55 (0.72-3.35) |  |  |  |  |  |  |  |  |
| P-trend |  |  | 0.153 |  |  |  |  |  |  |  |  |
| per 2 sizes | 33880 | 54 | 1.14 (0.83-1.58) |  |  |  |  |  |  |  |  |
|  | | | | | | | | | | | |
| Abbreviations: HR, hazard ratio; CI, confidence interval; CRC, colorectal cancer; (d)MMR, mismatch repair (deficient); NLCS, Netherlands Cohort Study; mut, mutated; BMI, body mass index.  ^a^Hazard Ratios were adjusted for age (years; continuous) and age was included as a time-varying covariate.  ^b^Analyses for subgroups with <50 cases were not performed. | | | | | | | | | | | |
|  | | | | | | | | | | | |

| **Supplementary Table S5 -** Age-adjusted HRs^a^ and 95%-CIs for associations between physical activity measures and CRC in subgroups based on mutation and MMR status, by sex and tumor location; NLCS, 1986-2006. | | | | | | | | | | | | | | |  |
| --- | --- | --- | --- | --- | --- | --- | --- | --- | --- | --- | --- | --- | --- | --- | --- |
|  | **Person-years at risk** | **Total** | | | | **Wild-type+pMMR^b^** | | | | | **Any-mutation/dMMR^c^** | | | | |
|  |  | n_cases_ | HR (95%-CI) | | | n_cases_ | HR (95%-CI) | | | | n_cases_ | HR (95%-CI) | | | |
| **Non-occupational physical activity (min/day): range (median)** | | | | |  | | |  |  | | | |  |  |  |
|  |  |  |  | | |  |  | | | |  |  | | | |
| **Men – colon** |  |  |  | | |  |  | | | |  |  | | | |
| ≤30 (21.4) | 4997 | 132 | 1.00 (ref.) | | | 49 | 1.00 (ref.) | | | | 83 | 1.00 (ref.) | | | |
| 31-60 (42.9) | 10100 | 242 | 0.87 (0.67-1.13) | | | 94 | 0.91 (0.63-1.32) | | | | 148 | 0.85 (0.63-1.16) | | | |
| 61-90 (73.6) | 6001 | 156 | 0.96 (0.73-1.28) | | | 62 | 1.02 (0.68-1.53) | | | | 94 | 0.93 (0.66-1.30) | | | |
| >90 (130.0) | 9925 | 224 | 0.80 (0.62-1.04) | | | 104 | 1.01 (0.70-1.47) | | | | 120 | 0.68 (0.49-0.93) | | | |
| P-trend |  |  | 0.169 | | |  | 0.676 | | | |  | 0.025 | | | |
| per 30 min/day | 31022 | 754 | 0.98 (0.94-1.02) | | | 309 | 1.01 (0.96-1.06) | | | | 445 | 0.96 (0.91-1.01) | | | |
| **Men – rectum** |  |  |  | | |  |  | | | |  |  | | | |
| ≤30 (21.4) | 4997 | 18 | 1.00 (ref.) | | | 13 | 1.00 (ref.) | | | | 5 | 1.00 (ref.) | | | |
| 31-60 (42.9) | 10100 | 73 | 1.95 (1.14-3.33) | | | 41 | 1.51 (0.80-2.86) | | | | 32 | 3.09 (1.19-8.04) | | | |
| 61-90 (73.6) | 6001 | 57 | 2.56 (1.47-4.46) | | | 38 | 2.34 (1.22-4.49) | | | | 19 | 3.12 (1.15-8.48) | | | |
| >90 (130.0) | 9925 | 76 | 2.08 (1.22-3.55) | | | 43 | 1.65 (0.87-3.13) | | | | 33 | 3.20 (1.23-8.32) | | | |
| P-trend |  |  | 0.015 | | |  | 0.097 | | | |  | 0.051 | | | |
| per 30 min/day | 31022 | 224 | 1.03 (0.98-1.09) | | | 135 | 1.04 (0.97-1.12) | | | | 89 | 1.02 (0.95-1.10) | | | |
| **Women – colon** |  |  |  | | |  |  | | | |  |  | | | |
| ≤30 (19.3) | 7756 | 169 | 1.00 (ref.) | | | 52 | 1.00 (ref.) | | | | 117 | 1.00 (ref.) | | | |
| 31-60 (42.9) | 10923 | 198 | 0.84 (0.66-1.07) | | | 44 | 0.59 (0.39-0.91) | | | | 154 | 0.95 (0.72-1.25) | | | |
| 61-90 (75.0) | 8000 | 148 | 0.86 (0.66-1.12) | | | 47 | 0.86 (0.57-1.31) | | | | 101 | 0.86 (0.64-1.17) | | | |
| >90 (115.7) | 7550 | 115 | 0.71 (0.54-0.94) | | | 36 | 0.70 (0.45-1.09) | | | | 79 | 0.72 (0.52-0.99) | | | |
| P-trend |  |  | 0.028 | | |  | 0.352 | | | |  | 0.033 | | | |
| per 30 min/day | 34228 | 630 | 0.97 (0.91-1.03) | | | 179 | 0.98 (0.88-1.09) | | | | 451 | 0.96 (0.90-1.03) | | | |
| **Women – rectum** |  |  |  | | |  |  | | | |  |  | | | |
| ≤30 (19.3) | 7756 | 31 | 1.00 (ref.) | | | 14 | 1.00 (ref.) | | | | 17 | 1.00 (ref.) | | | |
| 31-60 (42.9) | 10923 | 44 | 1.01 (0.63-1.62) | | | 26 | 1.29 (0.67-2.51) | | | | 18 | 0.76 (0.39-1.49) | | | |
| 61-90 (75.0) | 8000 | 34 | 1.06 (0.64-1.76) | | | 12 | 0.81 (0.36-1.78) | | | | 22 | 1.29 (0.68-2.45) | | | |
| >90 (115.7) | 7550 | 22 | 0.73 (0.42-1.28) | | | 12 | 0.85 (0.39-1.88) | | | | 10 | 0.63 (0.28-1.39) | | | |
| P-trend |  |  | 0.341 | | |  | 0.379 | | | |  | 0.625 | | | |
| per 30 min/day | 34228 | 131 | 1.01 (0.89-1.15) | | | 64 | 1.08 (0.91-1.28) | | | | 67 | 0.93 (0.80-1.09) | | | |
|  |  |  |  | | |  |  | | | |  |  | | | |
| **Occupational energy expenditure (kJ/min)** | | | |  |  | | |  |  | | | |  |  |  |
|  |  |  |  | | |  |  | | |  | |  | | | |
| **Men – colon** | 25073 | 564 |  | | | 226 |  | | | 338 | |  | | | |
| < 8 | 15144 | 365 | 1.00 (ref.) | | | 152 | 1.00 (ref.) | | | 213 | | 1.00 (ref.) | | | |
| 8-12 | 6368 | 133 | 0.86 (0.68-1.09) | | | 54 | 0.85 (0.61-1.18) | | | 79 | | 0.87 (0.65-1.16) | | | |
| >12 | 3561 | 66 | 0.75 (0.55-1.01) | | | 20 | 0.55 (0.34-0.90) | | | 46 | | 0.89 (0.62-1.26) | | | |
| P-trend |  |  | 0.038 | | |  | 0.014 | | |  | | 0.361 | | | |
| **Men – rectum** | 25073 | 185 |  | | | 114 |  | | | 71 | |  | | | |
| < 8 | 15144 | 107 | 1.00 (ref.) | | | 65 | 1.00 (ref.) | | | 42 | | 1.00 (ref.) | | | |
| 8-12 | 6368 | 57 | 1.28 (0.91-1.80) | | | 35 | 1.31 (0.85-2.00) | | | 22 | | 1.25 (0.74-2.11) | | | |
| >12 | 3561 | 21 | 0.83 (0.51-1.36) | | | 14 | 0.92 (0.51-1.68) | | | 7 | | 0.69 (0.31-1.55) | | | |
| P-trend |  |  | 0.962 | | |  | 0.788 | | |  | | 0.659 | | | |
|  |  |  |  | | |  |  | | |  | |  | | | |
| **Occupational sitting time (hours/day)** | | | | | | | | | | | | | | | |
|  |  |  |  | | |  |  | | |  | |  | | | |
| **Men – colon** | 25073 | 564 |  | | | 226 |  | | | 338 | |  | | | |
| > 6 | 6511 | 187 | 1.00 (ref.) | | | 85 | 1.00 (ref.) | | | 102 | | 1.00 (ref.) | | | |
| 2-6 | 11617 | 244 | 0.71 (0.57-0.89) | | | 87 | 0.56 (0.41-0.77) | | | 157 | | 0.83 (0.63-1.10) | | | |
| < 2 | 6944 | 133 | 0.66 (0.51-0.85) | | | 54 | 0.59 (0.41-0.85) | | | 79 | | 0.71 (0.51-0.99) | | | |
| P-trend |  |  | 0.002 | | |  | 0.005 | | |  | | 0.041 | | | |
| **Men – rectum** | 25073 | 185 |  | | | 114 |  | | | 71 | |  | | | |
| > 6 | 6511 | 60 | 1.00 (ref.) | | | 39 | 1.00 (ref.) | | | 21 | | 1.00 (ref.) | | | |
| 2-6 | 11617 | 69 | 0.64 (0.44-0.92) | | | 40 | 0.57 (0.36-0.90) | | | 29 | | 0.75 (0.42-1.33) | | | |
| < 2 | 6944 | 56 | 0.88 (0.59-1.29) | | | 35 | 0.85 (0.53-1.37) | | | 21 | | 0.93 (0.50-1.71) | | | |
| P-trend |  |  | 0.526 | | |  | 0.518 | | |  | | 0.825 | | | |
|  |  |  |  | | |  |  | | |  | |  | | | |
| Abbreviations: HR, hazard ratio; CI, confidence interval; CRC, colorectal cancer; (d/p)MMR, mismatch repair (deficient/proficient); NLCS, Netherlands Cohort Study.  ^a^Hazard Ratios were adjusted for age (years; continuous) and age was included as a time-varying covariate.  ^b^This group excludes cases with mutations in any of the genes (*KRAS*, *BRAF*, or *PIK3CA*), as well as MMR deficient cases.  ^c^This group includes cases with mutations in any of the genes (*KRAS*, *BRAF*, or *PIK3CA*) and/or cases that are MMR deficient. | | | | | | | | | | | | | | |  |

| **Supplementary Table S6 -** Age-adjusted HRs^a^ and 95%-CIs for associations between adiposity measures and CRC in for individual mutations and MMR status, by sex and tumor location; NLCS, 1986-2006. | | | | | | | | | | | | | | | | | | | | | | |  |
| --- | --- | --- | --- | --- | --- | --- | --- | --- | --- | --- | --- | --- | --- | --- | --- | --- | --- | --- | --- | --- | --- | --- | --- |
|  | **Person-years at risk** | ***KRAS*_mut_** | | ***PIK3CA*_mut_^b^** | | | | | | | ***BRAF*_mut_^b^** | | | | | **dMMR^b^** | | | | |  |  |  |
|  |  | n_cases_ | HR (95%-CI) | | n_cases_ | | | HR (95%-CI) | | | | n_cases_ | | | HR (95%-CI) | | n_cases_ | | HR (95%-CI) | | |  |  |
| **Non-occupational physical activity (min/day): range (median)** | | | | | | | | | | | | | |  |  | | |  | |  | |  |  |
|  |  |  |  | |  | | |  | | | |  | | |  | |  | |  | | |  |  |
| **Men – colon** | |  |  | | |  | | |  | | | |  | |  | | |  | |  | |  |  |
| ≤30 (21.4) | 4997 | 41 | 1.00 (ref.) | | 24 | | | 1.00 (ref.) | | | | 19 | | | 1.00 (ref.) | | 13 | | 1.00 (ref.) | | |  |  |
| 31-60 (42.9) | 10100 | 83 | 0.97 (0.65-1.45) | | 63 | | | 1.26 (0.77-2.07) | | | | 34 | | | 0.85 (0.47-1.52) | | 18 | | 0.65 (0.31-1.36) | | |  |  |
| 61-90 (73.6) | 6001 | 63 | 1.27 (0.83-1.94) | | 28 | | | 0.96 (0.55-1.70) | | | | 19 | | | 0.82 (0.43-1.58) | | 16 | | 1.02 (0.48-2.15) | | |  |  |
| >90 (130.0) | 9925 | 69 | 0.79 (0.52-1.20) | | 35 | | | 0.70 (0.41-1.21) | | | | 33 | | | 0.80 (0.44-1.45) | | 23 | | 0.79 (0.39-1.61) | | |  |  |
| P-trend |  |  | 0.324 | |  | | | 0.029 | | | |  | | | 0.515 | |  | | 0.934 | | |  |  |
| per 30 min/day | 31022 | 256 | 0.94 (0.88-1.00) | | 150 | | | 0.96 (0.87-1.06) | | | | 105 | | | 1.02 (0.93-1.11) | | 70 | | 1.02 (0.92-1.14) | | |  |  |
| **Men – rectum** | |  |  | | |  | | |  | | | |  | |  | | |  | |  | |  |  |
| ≤30 (21.4) | 4997 | 5 | 1.00 (ref.) | |  | | |  | | | |  | | |  | |  | |  | | |  |  |
| 31-60 (42.9) | 10100 | 26 | 2.50 (0.95-6.61) | |  | | |  | | | |  | | |  | |  | |  | | |  |  |
| 61-90 (73.6) | 6001 | 16 | 2.64 (0.95-7.32) | |  | | |  | | | |  | | |  | |  | |  | | |  |  |
| >90 (130.0) | 9925 | 21 | 2.00 (0.74-5.38) | |  | | |  | | | |  | | |  | |  | |  | | |  |  |
| P-trend |  |  | 0.464 | |  | | |  | | | |  | | |  | |  | |  | | |  |  |
| per 30 min/day | 31022 | 68 | 0.98 (0.88-1.08) | |  | | |  | | | |  | | |  | |  | |  | | |  |  |
| **Women – colon** | |  |  | | |  | | |  | | | |  | |  | | |  | |  | |  |  |
| ≤30 (19.3) | 7756 | 55 | 1.00 (ref.) | | 36 | | | 1.00 (ref.) | | | | 47 | | | 1.00 (ref.) | | 32 | | 1.00 (ref.) | | |  |  |
| 31-60 (42.9) | 10923 | 78 | 1.02 (0.70-1.48) | | 35 | | | 0.70 (0.43-1.14) | | | | 59 | | | 0.91 (0.61-1.36) | | 45 | | 1.02 (0.63-1.63) | | |  |  |
| 61-90 (75.0) | 8000 | 48 | 0.87 (0.57-1.31) | | 28 | | | 0.78 (0.46-1.30) | | | | 35 | | | 0.75 (0.47-1.19) | | 34 | | 1.05 (0.64-1.74) | | |  |  |
| >90 (115.7) | 7550 | 41 | 0.79 (0.51-1.21) | | 17 | | | 0.50 (0.28-0.91) | | | | 32 | | | 0.73 (0.45-1.18) | | 20 | | 0.66 (0.37-1.18) | | |  |  |
| P-trend |  |  | 0.192 | |  | | | 0.040 | | | |  | | | 0.131 | |  | | 0.207 | | |  |  |
| per 30 min/day | 34228 | 222 | 0.98 (0.89-1.08) | | 116 | | | 0.89 (0.76-1.05) | | | | 173 | | | 0.95 (0.85-1.05) | | 131 | | 0.98 (0.87-1.10) | | |  |  |
| **Women – rectum** | |  |  | | |  | | |  | | | |  | |  | | |  | |  | |  |  |
| ≤30 (19.3) | 7756 | 14 | 1.00 (ref.) | |  | | |  | | | |  | | |  | |  | |  | | |  |  |
| 31-60 (42.9) | 10923 | 15 | 0.77 (0.37-1.60) | |  | | |  | | | |  | | |  | |  | |  | | |  |  |
| 61-90 (75.0) | 8000 | 20 | 1.40 (0.71-2.79) | |  | | |  | | | |  | | |  | |  | |  | | |  |  |
| >90 (115.7) | 7550 | 6 | 0.45 (0.17-1.18) | |  | | |  | | | |  | | |  | |  | |  | | |  |  |
| P-trend |  |  | 0.388 | |  | | |  | | | |  | | |  | |  | |  | | |  |  |
| per 30 min/day | 34228 | 55 | 0.86 (0.74-1.00) | |  | | |  | | | |  | | |  | |  | |  | | |  |  |
|  |  |  |  | |  | | |  | | | |  | | |  | |  | |  | | |  |  |
| **Occupational energy expenditure (kJ/min)** | | | | | | |  | | |  | | | |  |  | | |  | |  | |  |  |
|  |  |  |  | |  | | |  | | | |  | | |  | |  | |  | | |  |  |
| **Men – colon** | 25073 | 190 |  | | 114 | | |  | | | | 87 | | |  | | | 58 | |  | |  |  |
| < 8 | 15144 | 115 | 1.00 (ref.) | | 72 | | | 1.00 (ref.) | | | | 59 | | | 1.00 (ref.) | | 32 | | 1.00 (ref.) | | |  |  |
| 8-12 | 6368 | 50 | 1.02 (0.72-1.45) | | 26 | | | 0.85 (0.54-1.35) | | | | 18 | | | 0.71 (0.41-1.23) | | 16 | | 1.14 (0.61-2.11) | | |  |  |
| >12 | 3561 | 25 | 0.89 (0.56-1.41) | | 16 | | | 0.92 (0.52-1.62) | | | | 10 | | | 0.69 (0.35-1.37) | | 10 | | 1.26 (0.60-2.63) | | |  |  |
| P-trend |  |  | 0.709 | |  | | | 0.625 | | | |  | | | 0.177 | |  | | 0.509 | | |  |  |
| **Men – rectum** | 25073 | 53 |  | |  | | |  | | | |  | | |  | |  | |  | | |  |  |
| < 8 | 15144 | 31 | 1.00 (ref.) | |  | | |  | | | |  | | |  | |  | |  | | |  |  |
| 8-12 | 6368 | 17 | 1.30 (0.71-2.36) | |  | | |  | | | |  | | |  | | |  | |  | |  |  |
| >12 | 3561 | 5 | 0.66 (0.26-1.70) | |  | | |  | | | |  | | |  | |  | |  | | |  |  |
| P-trend |  |  | 0.686 | |  | | |  | | | |  | | |  | |  | |  | | |  |  |
|  |  |  |  | |  | | |  | | | |  | | |  | |  | |  | | |  |  |
| **Occupational sitting time (hours/day)** | | | | | | | | | | | | | | | | | | | | | | |  |
|  |  |  |  | |  | | |  | | | |  | | |  | |  | |  | | |  |  |
| **Men – colon** | 25073 | 190 |  | | 114 | | |  | | | | 87 | | |  | | 58 | |  | | |  |  |
| > 6 | 6511 | 57 | 1.00 (ref.) | | 34 | | | 1.00 (ref.) | | | | 22 | | | 1.00 (ref.) | | | 14 | | 1.00 (ref.) | |  |  |
| 2-6 | 11617 | 87 | 0.83 (0.58-1.18) | | 48 | | | 0.77 (0.49-1.21) | | | | 47 | | | 1.15 (0.68-1.94) | | 27 | | 1.02 (0.52-1.98) | | |  |  |
| < 2 | 6944 | 46 | 0.74 (0.49-1.12) | | 32 | | | 0.87 (0.53-1.44) | | | | 18 | | | 0.75 (0.40-1.42) | | 17 | | 1.09 (0.52-2.25) | | |  |  |
| P-trend |  |  | 0.156 | |  | | | 0.602 | | | |  | | | 0.352 | |  | | 0.819 | | |  |  |
| **Men – rectum** | 25073 | 53 |  | |  | | |  | | | |  | | |  | |  | |  | | |  |  |
| > 6 | 6511 | 17 | 1.00 (ref.) | |  | | |  | | | |  | | |  | |  | |  | | |  |  |
| 2-6 | 11617 | 20 | 0.63 (0.33-1.22) | |  | | |  | | | |  | | |  | |  | |  | | |  |  |
| < 2 | 6944 | 16 | 0.86 (0.43-1.72) | |  | | |  | | | |  | | |  | | |  | |  | |  |  |
| P-trend |  |  | 0.700 | |  | | |  | | | |  | | |  | |  | |  | | |  |  |
|  | | | | | | | | | | | | | | | | | | | | | | | |
| Abbreviations: HR, hazard ratio; CI, confidence interval; CRC, colorectal cancer; (d)MMR, mismatch repair (deficient); NLCS, Netherlands Cohort Study; mut, mutated.  ^a^Hazard Ratios were adjusted for age (years; continuous) and age was included as a time-varying covariate.  ^b^Analyses for subgroups with <50 cases were not performed. | | | | | | | | | | | | | | | | | | | | | | | |
|  | | | | | | | | | | | | | | | | | | | | | | | |

| **Supplementary Table S7 -** Multivariable-adjusted HRs^a^ and 95%-CIs for associations between adiposity measures and CRC subgroups based on individual mutation and MMR status, by sex and tumor location; NLCS, 1986-2006. | | | | | | | | | |
| --- | --- | --- | --- | --- | --- | --- | --- | --- | --- |
|  | **Person-years at risk** | ***KRAS*_wt_** | | ***PIK3CA*_wt_^b^** | | ***BRAF*_wt_^b^** | | **pMMR^b^** | |
|  |  | n_cases_ | HR (95%-CI) | n_cases_ | HR (95%-CI) | n_cases_ | HR (95%-CI) | n_cases_ | HR (95%-CI) |
| **BMI quartiles (kg/m^2^): range (median)** | | | |  |  |  |  |  |  |
|  |  |  |  |  |  |  |  |  |  |
| **Men – colon** | |  |  |  |  |  |  |  |  |
| < 23.4 (22.2) | 7993 | 103 | 1.00 (ref.) | 140 | 1.00 (ref.) | 142 | 1.00 (ref.) | 148 | 1.00 (ref.) |
| 23.4-24.9 (24.2) | 8343 | 130 | 1.16 (0.87-1.57) | 150 | 1.00 (0.76-1.32) | 160 | 1.06 (0.81-1.38) | 175 | 1.11 (0.85-1.44) |
| 25.0-26.6 (25.7) | 7683 | 131 | 1.26 (0.93-1.71) | 158 | 1.12 (0.85-1.48) | 171 | 1.21 (0.92-1.59) | 179 | 1.21 (0.93-1.59) |
| > 26.6 (27.8) | 7003 | 131 | 1.47 (1.09-1.99) | 156 | 1.30 (0.99-1.72) | 176 | 1.46 (1.11-1.91) | 182 | 1.44 (1.10-1.89) |
| P-trend |  |  | 0.011 |  | 0.044 |  | 0.004 |  | 0.006 |
| per 5 kg/m^2^ | 31022 | 498 | 1.33 (1.11-1.58) | 604 | 1.28 (1.09-1.51) | 649 | 1.32 (1.12-1.54) | 684 | 1.28 (1.10-1.50) |
| **Men – rectum** | |  |  |  |  |  |  |  |  |
| < 23.4 (22.2) | 7993 | 40 | 1.00 (ref.) |  |  |  |  |  |  |
| 23.4-24.9 (24.2) | 8343 | 41 | 0.94 (0.59-1.49) |  |  |  |  |  |  |
| 25.0-26.6 (25.7) | 7683 | 46 | 1.18 (0.75-1.85) |  |  |  |  |  |  |
| > 26.6 (27.8) | 7003 | 29 | 0.81 (0.49-1.36) |  |  |  |  |  |  |
| P-trend |  |  | 0.706 |  |  |  |  |  |  |
| per 5 kg/m^2^ | 31022 | 156 | 0.96 (0.73-1.26) |  |  |  |  |  |  |
| **Women – colon** | |  |  |  |  |  |  |  |  |
| <22.8 (21.5) | 9014 | 129 | 1.00 (ref.) | 151 | 1.00 (ref.) | 128 | 1.00 (ref.) | 138 | 1.00 (ref.) |
| 22.8-24.7 (23.8) | 8914 | 100 | 0.78 (0.58-1.05) | 119 | 0.80 (0.60-1.05) | 106 | 0.83 (0.62-1.11) | 112 | 0.82 (0.62-1.09) |
| 24.8-27.0 (25.7) | 8141 | 82 | 0.71 (0.52-0.98) | 117 | 0.89 (0.67-1.19) | 108 | 0.97 (0.72-1.30) | 123 | 1.03 (0.77-1.37) |
| >27.0 (29.2) | 8158 | 97 | 0.86 (0.63-1.18) | 127 | 1.01 (0.76-1.34) | 115 | 1.05 (0.78-1.42) | 126 | 1.08 (0.81-1.44) |
| P-trend |  |  | 0.255 |  | 0.856 |  | 0.583 |  | 0.393 |
| per 5 kg/m^2^ | 34228 | 408 | 0.90 (0.77-1.05) | 514 | 1.03 (0.90-1.18) | 457 | 1.06 (0.91-1.22) | 499 | 1.08 (0.94-1.24) |
| **Women – rectum** | |  |  |  |  |  |  |  |  |
| <22.8 (21.5) | 9014 | 19 | 1.00 (ref.) |  |  |  |  |  |  |
| 22.8-24.7 (23.8) | 8914 | 19 | 0.96 (0.49-1.87) |  |  |  |  |  |  |
| 24.8-27.0 (25.7) | 8141 | 23 | 1.25 (0.66-2.37) |  |  |  |  |  |  |
| >27.0 (29.2) | 8158 | 15 | 0.78 (0.39-1.58) |  |  |  |  |  |  |
| P-trend |  |  | 0.714 |  |  |  |  |  |  |
| per 5 kg/m^2^ | 34228 | 76 | 0.99 (0.74-1.33) |  |  |  |  |  |  |
|  |  |  |  |  |  |  |  |  |  |
| **Clothing size: range (median)** | | | | | | | | | |
|  |  |  |  |  |  |  |  |  |  |
| **Men – colon** | |  |  |  |  |  |  |  |  |
| ≤50 (50) | 10903 | 138 | 1.00 (ref.) | 171 | 1.00 (ref.) | 181 | 1.00 (ref.) | 193 | 1.00 (ref.) |
| 52 (52) | 9750 | 163 | 1.31 (1.01-1.71) | 195 | 1.26 (0.99-1.61) | 218 | 1.34 (1.06-1.69) | 224 | 1.29 (1.02-1.63) |
| 54 (54) | 5156 | 88 | 1.36 (1.00-1.86) | 114 | 1.39 (1.04-1.85) | 115 | 1.35 (1.01-1.79) | 124 | 1.36 (1.03-1.79) |
| ≥56 (56) | 2619 | 61 | 1.89 (1.32-2.70) | 73 | 1.79 (1.28-2.52) | 81 | 1.90 (1.37-2.64) | 83 | 1.83 (1.32-2.53) |
| P-trend |  |  | 0.001 |  | <0.001 |  | <0.001 |  | <0.001 |
| per 2 sizes | 28428 | 450 | 1.37 (1.18-1.60) | 553 | 1.33 (1.15-1.54) | 595 | 1.35 (1.17-1.55) | 624 | 1.33 (1.16-1.52) |
| **Men – rectum** | |  |  |  |  |  |  |  |  |
| ≤50 (50) | 10903 | 57 | 1.00 (ref.) |  |  |  |  |  |  |
| 52 (52) | 9750 | 52 | 1.03 (0.69-1.53) |  |  |  |  |  |  |
| 54 (54) | 5156 | 26 | 0.99 (0.61-1.62) |  |  |  |  |  |  |
| ≥56 (56) | 2619 | 7 | 0.55 (0.25-1.23) |  |  |  |  |  |  |
| P-trend |  |  | 0.293 |  |  |  |  |  |  |
| per 2 sizes | 28428 | 142 | 0.91 (0.73-1.13) |  |  |  |  |  |  |
| **Women – colon** | |  |  |  |  |  |  |  |  |
| ≤40 (40) | 6574 | 93 | 1.00 (ref.) | 104 | 1.00 (ref.) | 99 | 1.00 (ref.) | 96 | 1.00 (ref.) |
| 42 (42) | 8582 | 96 | 0.79 (0.57-1.10) | 125 | 0.92 (0.68-1.24) | 103 | 0.78 (0.57-1.07) | 115 | 0.90 (0.66-1.23) |
| 44 (44) | 9270 | 101 | 0.74 (0.53-1.01) | 126 | 0.83 (0.62-1.12) | 114 | 0.78 (0.57-1.06) | 134 | 0.94 (0.70-1.28) |
| ≥46 (46) | 9454 | 112 | 0.81 (0.59-1.12) | 152 | 1.01 (0.75-1.36) | 133 | 0.91 (0.67-1.23) | 145 | 1.02 (0.76-1.38) |
| P-trend |  |  | 0.230 |  | 0.994 |  | 0.701 |  | 0.747 |
| per 2 sizes | 33880 | 402 | 1.00 (0.85-1.18) | 507 | 1.09 (0.94-1.27) | 449 | 1.10 (0.94-1.29) | 490 | 1.14 (0.98-1.32) |
| **Women – rectum** | |  |  |  |  |  |  |  |  |
| ≤40 (40) | 6574 | 13 | 1.00 (ref.) |  |  |  |  |  |  |
| 42 (42) | 8582 | 21 | 1.16 (0.56-2.40) |  |  |  |  |  |  |
| 44 (44) | 9270 | 23 | 1.23 (0.61-2.45) |  |  |  |  |  |  |
| ≥46 (46) | 9454 | 19 | 0.94 (0.45-1.95) |  |  |  |  |  |  |
| P-trend |  |  | 0.835 |  |  |  |  |  |  |
| per 2 sizes | 33880 | 76 | 0.94 (0.72-1.22) |  |  |  |  |  |  |
|  | | | | | | | | | |
| Abbreviations: HR, hazard ratio; CI, confidence interval; CRC, colorectal cancer; (d/p)MMR, mismatch repair (deficient/proficient);; NLCS, Netherlands Cohort Study; wt, wild-type.  ^a^Hazard Ratios were adjusted for age (years; continuous), non-occupational physical activity (minutes/day; continuous), total energy intake (kcal/day; continuous), family history of CRC (yes/no), alcohol consumption (0; 0.1-4; 5-14; >15 g/day), processed meat intake (g/day; continuous), red meat intake (g/day; continuous). Age was included as a time-varying covariate. BMI models were additionally adjusted for height (cm; continuous).  ^b^Analyses were not performed when <50 cases showed a mutation or dMMR. | | | | | | | | | |
|  | | | | | | | | | |

| **Supplementary Table S8 –** Multivariable-adjusted HRs^a^ and 95%-CIs for associations between physical activity measures and CRC subgroups based on individual mutation and MMR status, by sex and tumor location; NLCS, 1986-2006. | | | | | | | | | | |
| --- | --- | --- | --- | --- | --- | --- | --- | --- | --- | --- |
|  | **Person-years at risk** | | ***KRAS*_wt_** | | ***PIK3CA*_wt_^b^** | | ***BRAF*_wt_^b^** | | **pMMR^b^** | |
|  |  |  | n_cases_ | HR (95%-CI) | n_cases_ | HR (95%-CI) | n_cases_ | HR (95%-CI) | n_cases_ | HR (95%-CI) |
| **Non-occupational physical activity (min/day) : range (median)** | | | | | | |  |  |  |  |
|  |  | |  |  |  |  |  |  |  |  |
| **Men – colon** | | |  |  |  |  |  |  |  |  |
| ≤30 (21.4) | 4997 | | 91 | 1.00 (ref.) | 108 | 1.00 (ref.) | 113 | 1.00 (ref.) | 119 | 1.00 (ref.) |
| 31-60 (42.9) | 10100 | | 159 | 0.86 (0.63-1.16) | 179 | 0.80 (0.60-1.07) | 208 | 0.90 (0.68-1.18) | 224 | 0.92 (0.70-1.20) |
| 61-90 (73.6) | 6001 | | 93 | 0.85 (0.61-1.19) | 128 | 1.00 (0.74-1.36) | 137 | 1.02 (0.76-1.38) | 140 | 1.00 (0.74-1.34) |
| >90 (130.0) | 9925 | | 155 | 0.85 (0.63-1.15) | 189 | 0.87 (0.66-1.16) | 191 | 0.85 (0.64-1.13) | 201 | 0.86 (0.65-1.13) |
| P-trend |  | |  | 0.414 |  | 0.838 |  | 0.422 |  | 0.346 |
| per 30 min/day | | 31022 | 498 | 1.01 (0.96-1.06) | 604 | 1.00 (0.95-1.04) | 649 | 0.98 (0.94-1.03) | 684 | 0.99 (0.94-1.03) |
| **Men – rectum** | | |  |  |  |  |  |  |  |  |
| ≤30 (21.4) | 4997 | | 13 | 1.00 (ref.) |  |  |  |  |  |  |
| 31-60 (42.9) | 10100 | | 47 | 1.72 (0.91-3.26) |  |  |  |  |  |  |
| 61-90 (73.6) | 6001 | | 41 | 2.52 (1.32-4.81) |  |  |  |  |  |  |
| >90 (130.0) | 9925 | | 55 | 2.12 (1.13-3.97) |  |  |  |  |  |  |
| P-trend |  | |  | 0.012 |  |  |  |  |  |  |
| per 30 min/day | 31022 | | 156 | 1.06 (0.99-1.12) |  |  |  |  |  |  |
| **Women – colon** | | |  |  |  |  |  |  |  |  |
| ≤30 (19.3) | 7756 | | 114 | 1.00 (ref.) | 133 | 1.00 (ref.) | 122 | 1.00 (ref.) | 137 | 1.00 (ref.) |
| 31-60 (42.9) | 10923 | | 120 | 0.74 (0.56-1.00) | 163 | 0.86 (0.66-1.12) | 139 | 0.80 (0.61-1.06) | 153 | 0.79 (0.60-1.04) |
| 61-90 (75.0) | 8000 | | 100 | 0.84 (0.62-1.14) | 120 | 0.85 (0.64-1.14) | 113 | 0.88 (0.66-1.18) | 114 | 0.80 (0.60-1.07) |
| >90 (115.7) | 7550 | | 74 | 0.66 (0.47-0.91) | 98 | 0.75 (0.56-1.01) | 83 | 0.70 (0.51-0.96) | 95 | 0.72 (0.53-0.97) |
| P-trend |  | |  | 0.036 |  | 0.078 |  | 0.063 |  | 0.044 |
| per 30 min/day | 34228 | | 408 | 0.96 (0.89-1.03) | 514 | 0.98 (0.92-1.05) | 457 | 0.98 (0.91-1.05) | 499 | 0.97 (0.90-1.03) |
| **Women – rectum** | | |  |  |  |  |  |  |  |  |
| ≤30 (19.3) | 7756 | | 17 | 1.00 (ref.) |  |  |  |  |  |  |
| 31-60 (42.9) | 10923 | | 29 | 1.20 (0.65-2.24) |  |  |  |  |  |  |
| 61-90 (75.0) | 8000 | | 14 | 0.78 (0.38-1.60) |  |  |  |  |  |  |
| >90 (115.7) | 7550 | | 16 | 0.92 (0.46-1.86) |  |  |  |  |  |  |
| P-trend |  | |  | 0.495 |  |  |  |  |  |  |
| per 30 min/day | 34228 | | 76 | 1.08 (0.93-1.26) |  |  |  |  |  |  |
|  |  | |  |  |  |  |  |  |  |  |
| **Occupational energy expenditure (kJ/min)** | | | | |  |  |  |  |  |  |
|  | | | | |  |  |  |  |  |  |
|  | | |  |  |  |  |  |  |  |  |
| **Men – colon** | 25073 | | 374 |  | 450 |  | 477 |  | 506 |  |
| < 8 | 15144 | | 250 | 1.00 (ref.) | 293 | 1.00 (ref.) | 306 | 1.00 (ref.) | 333 | 1.00 (ref.) |
| 8-12 | 6368 | | 83 | 0.74 (0.56-0.98) | 107 | 0.83 (0.64-1.08) | 115 | 0.86 (0.67-1.11) | 117 | 0.80 (0.63-1.03) |
| >12 | 3561 | | 41 | 0.61 (0.42-0.89) | 50 | 0.67 (0.47-0.95) | 56 | 0.72 (0.51-1.01) | 56 | 0.67 (0.48-0.94) |
| P-trend |  | |  | 0.003 |  | 0.016 |  | 0.038 |  | 0.008 |
| **Men – rectum** | 25073 | | 132 |  |  |  |  |  |  |  |
| < 8 | 15144 | | 76 | 1.00 (ref.) |  |  |  |  |  |  |
| 8-12 | 6368 | | 40 | 1.31 (0.87-1.98) |  |  |  |  |  |  |
| > 12 | 3561 | | 16 | 0.89 (0.50-1.58) |  |  |  |  |  |  |
| P-trend |  | |  | 0.849 |  |  |  |  |  |  |
|  |  | |  |  |  |  |  |  |  |  |
| **Occupational sitting time (hours/day)** | | | | |  |  |  |  |  |  |
|  | | | | |  |  |  |  |  |  |
| **Men – colon** | 25073 | | 374 |  | 450 |  | 477 |  | 506 |  |
| > 6 | 6511 | | 130 | 1.00 (ref.) | 153 | 1.00 (ref.) | 165 | 1.00 (ref.) | 173 | 1.00 (ref.) |
| 2-6 | 11617 | | 157 | 0.65 (0.50-0.85) | 196 | 0.68 (0.53-0.88) | 197 | 0.64 (0.50-0.82) | 217 | 0.67 (0.53-0.86) |
| < 2 | 6944 | | 87 | 0.58 (0.43-0.80) | 101 | 0.59 (0.44-0.79) | 115 | 0.62 (0.47-0.82) | 116 | 0.60 (0.45-0.80) |
| P-trend |  | |  | 0.001 |  | <0.001 |  | 0.001 |  | <0.001 |
| **Men – rectum** | 25073 | | 132 |  |  |  |  |  |  |  |
| > 6 | 6511 | | 43 | 1.00 (ref.) |  |  |  |  |  |  |
| 2-6 | 11617 | | 49 | 0.62 (0.40-0.96) |  |  |  |  |  |  |
| < 2 | 6944 | | 40 | 0.87 (0.55-1.38) |  |  |  |  |  |  |
| P-trend |  | |  | 0.574 |  |  |  |  |  |  |
|  | | |  |  |  |  |  |  |  |  |
| Abbreviations: HR, hazard ratio; CI, confidence interval; CRC, colorectal cancer; (d/p)MMR, mismatch repair (deficient/proficient); NLCS, Netherlands Cohort Study; wt, wild-type.  ^a^Hazard Ratios were adjusted for age (years; continuous), BMI (kg/m^2^), total energy intake (kcal/day; continuous), family history of CRC (yes/no), alcohol consumption (0; 0.1-4; 5-14; >15 g/day), processed meat intake (g/day; continuous), red meat intake (g/day; continuous). Age was included as a time-varying covariate.  ^b^Analyses were not performed when <50 cases showed a mutation or dMMR. | | | | | | | | | | |

| **Supplementary Table S9 –** Multivariable-adjusted HRs^a^ and 95%-CIs for associations between (mutually adjusted) adiposity measures and CRC in subgroups based on mutation and MMR status, by sex and tumor location; NLCS, 1986-2006. | | | | | | | | | | | | | | |  |
| --- | --- | --- | --- | --- | --- | --- | --- | --- | --- | --- | --- | --- | --- | --- | --- |
|  | **Person-years at risk** | **Total** | | | | **Wild-type+pMMR^b^** | | | | | **Any-mutation/dMMR^c^** | | | | |
|  |  | n_cases_ | HR (95%-CI) | | | n_cases_ | HR (95%-CI) | | | | n_cases_ | HR (95%-CI) | | | |
| **BMI quartiles (kg/m^2^): range (median)** | | | |  |  | | |  |  | | | |  |  |  |
|  |  |  |  | | |  |  | | | |  |  | | | |
| **Men – colon** |  |  |  | | |  |  | | | |  |  | | | |
| < 23.4 (22.2) | 7356 | 153 | 1.00 (ref.) | | | 61 | 1.00 (ref.) | | | | 92 | 1.00 (ref.) | | | |
| 23.4-24.9 (24.2) | 7756 | 176 | 0.97 (0.73-1.27) | | | 73 | 0.95 (0.64-1.42) | | | | 103 | 0.98 (0.70-1.37) | | | |
| 25.0-26.6 (25.7) | 7056 | 184 | 1.01 (0.75-1.37) | | | 72 | 0.92 (0.61-1.41) | | | | 112 | 1.08 (0.75-1.56) | | | |
| > 26.6 (27.8) | 6260 | 171 | 1.02 (0.72-1.43) | | | 80 | 1.06 (0.67-1.68) | | | | 91 | 0.98 (0.65-1.50) | | | |
| P-trend |  |  | 0.836 | | |  | 0.842 | | | |  | 0.904 | | | |
| per 5 kg/m^2^ | 28428 | 684 | 1.16 (0.95-1.40) | | | 286 | 1.20 (0.91-1.58) | | | | 398 | 1.12 (0.89-1.42) | | | |
| **Men – rectum** |  |  |  | | |  |  | | | |  |  | | | |
| < 23.4 (22.2) | 7356 | 54 | 1.00 (ref.) | | | 30 | 1.00 (ref.) | | | | 24 | 1.00 (ref.) | | | |
| 23.4-24.9 (24.2) | 7756 | 51 | 0.82 (0.54-1.25) | | | 33 | 0.98 (0.58-1.66) | | | | 18 | 0.64 (0.34-1.21) | | | |
| 25.0-26.6 (25.7) | 7056 | 62 | 1.11 (0.72-1.70) | | | 40 | 1.35 (0.78-2.34) | | | | 22 | 0.82 (0.43-1.55) | | | |
| > 26.6 (27.8) | 6260 | 39 | 0.78 (0.46-1.31) | | | 21 | 0.79 (0.38-1.64) | | | | 18 | 0.74 (0.38-1.45) | | | |
| P-trend |  |  | 0.693 | | |  | 0.932 | | | |  | 0.570 | | | |
| per 5 kg/m^2^ | 28428 | 206 | 0.97 (0.73-1.30) | | | 124 | 0.95 (0.65-1.39) | | | | 82 | 1.01 (0.67-1.52) | | | |
| **Women – colon** |  |  |  | | |  |  | | | |  |  | | | |
| <22.8 (21.5) | 8964 | 179 | 1.00 (ref.) | | | 56 | 1.00 (ref.) | | | | 123 | 1.00 (ref.) | | | |
| 22.8-24.7 (23.8) | 8874 | 145 | 0.78 (0.58-1.05) | | | 43 | 0.73 (0.43-1.23) | | | | 102 | 0.80 (0.57-1.12) | | | |
| 24.8-27.0 (25.7) | 8101 | 143 | 0.84 (0.58-1.22) | | | 35 | 0.63 (0.29-1.39) | | | | 108 | 0.94 (0.63-1.39) | | | |
| >27.0 (29.2) | 7941 | 152 | 0.89 (0.52-1.51) | | | 44 | 0.75 (0.22-2.54) | | | | 108 | 0.95 (0.57-1.59) | | | |
| P-trend |  |  | 0.710 | | |  | 0.563 | | | |  | 0.987 | | | |
| per 5 kg/m^2^ | 33880 | 619 | 1.00 (0.78-1.28) | | | 178 | 0.70 (0.40-1.23) | | | | 441 | 1.15 (0.90-1.46) | | | |
| **Women – rectum** |  |  |  | | |  |  | | | |  |  | | | |
| <22.8 (21.5) | 8964 | 37 | 1.00 (ref.) | | | 16 | 1.00 (ref.) | | | | 21 | 1.00 (ref.) | | | |
| 22.8-24.7 (23.8) | 8874 | 26 | 0.80 (0.44-1.45) | | | 15 | 1.00 (0.41-2.40) | | | | 11 | 0.65 (0.29-1.41) | | | |
| 24.8-27.0 (25.7) | 8101 | 32 | 1.18 (0.62-2.24) | | | 18 | 1.37 (0.55-3.46) | | | | 14 | 1.03 (0.43-2.49) | | | |
| >27.0 (29.2) | 7941 | 35 | 1.58 (0.67-3.74) | | | 15 | 1.25 (0.34-4.55) | | | | 20 | 2.01 (0.66-6.16) | | | |
| P-trend |  |  | 0.230 | | |  | 0.562 | | | |  | 0.243 | | | |
| per 5 kg/m^2^ | 33880 | 130 | 1.40 (0.95-2.05) | | | 64 | 1.31 (0.75-2.28) | | | | 66 | 1.49 (0.89-2.48) | | | |
|  |  |  |  | | |  |  | | | |  |  | | | |
| **Clothing size: range (median)** | | | |  |  | | |  |  | | | |  |  |  |
|  |  |  |  | | |  |  | | |  | |  | | | |
| **Men – colon** |  |  |  | | |  |  | | |  | |  | | | |
| ≤50 (50) | 10903 | 211 | 1.00 (ref.) | | | 90 | 1.00 (ref.) | | | 121 | | 1.00 (ref.) | | | |
| 52 (52) | 9750 | 247 | 1.10 (0.86-1.41) | | | 104 | 1.17 (0.82-1.65) | | | 143 | | 1.06 (0.79-1.43) | | | |
| 54 (54) | 5156 | 136 | 1.05 (0.76-1.44) | | | 53 | 1.07 (0.68-1.67) | | | 83 | | 1.03 (0.71-1.51) | | | |
| ≥56 (56) | 2619 | 90 | 1.14 (0.74-1.76) | | | 39 | 1.37 (0.75-2.52) | | | 51 | | 1.00 (0.60-1.67) | | | |
| P-trend |  |  | 0.648 | | |  | 0.453 | | |  | | 0.985 | | | |
| per 2 sizes | 28428 | 684 | 1.13 (0.95-1.34) | | | 286 | 1.22 (0.96-1.54) | | | 398 | | 1.06 (0.86-1.31) | | | |
| **Men – rectum** |  |  |  | | |  |  | | |  | |  | | | |
| ≤50 (50) | 10903 | 78 | 1.00 (ref.) | | | 46 | 1.00 (ref.) | | | 32 | | 1.00 (ref.) | | | |
| 52 (52) | 9750 | 69 | 0.99 (0.68-1.45) | | | 46 | 1.07 (0.66-1.74) | | | 23 | | 0.86 (0.49-1.51) | | | |
| 54 (54) | 5156 | 43 | 1.20 (0.74-1.95) | | | 25 | 1.08 (0.59-2.00) | | | 18 | | 1.39 (0.69-2.82) | | | |
| ≥56 (56) | 2619 | 16 | 0.90 (0.45-1.82) | | | 7 | 0.59 (0.22-1.61) | | | 9 | | 1.47 (0.60-3.59) | | | |
| P-trend |  |  | 0.782 | | |  | 0.635 | | |  | | 0.274 | | | |
| per 2 sizes | 28428 | 206 | 0.95 (0.74-1.22) | | | 124 | 0.91 (0.66-1.26) | | | 82 | | 1.01 (0.73-1.41) | | | |
| **Women – colon** |  |  |  | | |  |  | | |  | |  | | | |
| ≤40 (40) | 6574 | 128 | 1.00 (ref.) | | | 46 | 1.00 (ref.) | | | 82 | | 1.00 (ref.) | | | |
| 42 (42) | 8582 | 150 | 0.82 (0.60-1.11) | | | 34 | 0.62 (0.37-1.03) | | | 116 | | 0.92 (0.65-1.31) | | | |
| 44 (44) | 9270 | 159 | 0.74 (0.52-1.05) | | | 48 | 0.85 (0.48-1.52) | | | 111 | | 0.71 (0.47-1.07) | | | |
| ≥46 (46) | 9454 | 182 | 0.79 (0.50-1.25) | | | 50 | 1.02 (0.47-2.18) | | | 132 | | 0.74 (0.44-1.24) | | | |
| P-trend |  |  | 0.270 | | |  | 0.778 | | |  | | 0.136 | | | |
| per 2 sizes | 33880 | 619 | 1.19 (0.91-1.57) | | | 178 | 1.62 (0.96-2.73) | | | 441 | | 1.05 (0.82-1.35) | | | |
| **Women – rectum** |  |  |  | | |  |  | | |  | |  | | | |
| ≤40 (40) | 6574 | 23 | 1.00 (ref.) | | | 11 | 1.00 (ref.) | | | 12 | | 1.00 (ref.) | | | |
| 42 (42) | 8582 | 30 | 0.89 (0.49-1.61) | | | 17 | 1.12 (0.49-2.52) | | | 13 | | 0.69 (0.30-1.61) | | | |
| 44 (44) | 9270 | 35 | 0.94 (0.50-1.77) | | | 20 | 1.23 (0.52-2.91) | | | 15 | | 0.69 (0.28-1.71) | | | |
| ≥46 (46) | 9454 | 42 | 1.06 (0.46-2.47) | | | 16 | 0.89 (0.24-3.33) | | | 26 | | 1.19 (0.40-3.52) | | | |
| P-trend |  |  | 0.831 | | |  | 0.943 | | |  | | 0.685 | | | |
| per 2 sizes | 33880 | 130 | 0.80 (0.54-1.17) | | | 64 | 0.89 (0.52-1.53) | | | 66 | | 0.72 (0.42-1.21) | | | |
|  |  |  |  | | |  |  | | |  | |  | | | |
| Abbreviations: HR, hazard ratio; CI, confidence interval; CRC, colorectal cancer; (d/p)MMR, mismatch repair (deficient/proficient); NLCS, Netherlands Cohort Study; BMI, body mass index.  ^a^Hazard Ratios were adjusted for age (years; continuous), non-occupational physical activity (minutes/day; continuous), total energy intake (kcal/day; continuous), family history of CRC (yes/no), alcohol consumption (0; 0.1-4; 5-14; >15 g/day), processed meat intake (g/day; continuous), red meat intake (g/day; continuous). Age was included as a time-varying covariate. BMI models were additionally adjusted for height (cm; continuous). Mutual adjustment: clothing-size (size; continuous) was included in BMI models, and BMI (kg/m^2^; continuous) was included in clothing-size models.  ^b^This group excludes cases with mutations in any of the genes (*KRAS*, *BRAF*, or *PIK3CA*), as well as MMR deficient cases.  ^c^This group includes cases with mutations in any of the genes (*KRAS*, *BRAF*, or *PIK3CA*) and/or cases that are MMR deficient. | | | | | | | | | | | | | | |  |

| **Supplementary Table S10 -** Multivariable-adjusted HRs^a^ and 95%-CIs for associations between (mutually adjusted) adiposity measures and CRC in for individual mutations and MMR status, by sex and tumor location; NLCS, 1986-2006. | | | | | | | | | | |  |
| --- | --- | --- | --- | --- | --- | --- | --- | --- | --- | --- | --- |
|  | **Person-years at risk** | ***KRAS*_mut_** | | ***PIK3CA*_mut_^a^** | | ***BRAF*_mut_^a^** | | **dMMR^a^** | |  |  |
|  |  | n_cases_ | HR (95%-CI) | n_cases_ | HR (95%-CI) | n_cases_ | HR (95%-CI) | n_cases_ | HR (95%-CI) |  |  |
| **BMI quartiles (kg/m^2^): range (median)** | | | |  |  |  |  |  |  |  |  |
|  |  |  |  |  |  |  |  |  |  |  |  |
| **Men – colon** | |  |  |  |  |  |  |  |  |  |  |
| < 23.4 (22.2) | 7993 | 56 | 1.00 (ref.) | 25 | 1.00 (ref.) | 22 | 1.00 (ref.) | 18 | 1.00 (ref.) |  |  |
| 23.4-24.9 (24.2) | 8343 | 56 | 0.89 (0.58-1.36) | 35 | 1.30 (0.74-2.30) | 27 | 1.12 (0.60-2.09) | 13 | 0.62 (0.29-1.31) |  |  |
| 25.0-26.6 (25.7) | 7683 | 62 | 1.00 (0.64-1.58) | 38 | 1.52 (0.84-2.78) | 26 | 1.09 (0.52-2.29) | 17 | 0.80 (0.36-1.80) |  |  |
| > 26.6 (27.8) | 7003 | 60 | 1.13 (0.68-1.87) | 33 | 1.49 (0.77-2.92) | 14 | 0.68 (0.27-1.72) | 12 | 0.61 (0.24-1.55) |  |  |
| P-trend |  |  | 0.539 |  | 0.200 |  | 0.505 |  | 0.457 |  |  |
| per 5 kg/m^2^ | 31022 | 234 | 1.19 (0.91-1.57) | 131 | 1.26 (0.88-1.82) | 89 | 0.99 (0.60-1.63) | 60 | 1.05 (0.61-1.83) |  |  |
| **Men – rectum** | |  |  |  |  |  |  |  |  |  |  |
| < 23.4 (22.2) | 7993 | 18 | 1.00 (ref.) |  |  |  |  |  |  |  |  |
| 23.4-24.9 (24.2) | 8343 | 13 | 0.57 (0.27-1.20) |  |  |  |  |  |  |  |  |
| 25.0-26.6 (25.7) | 7683 | 18 | 0.78 (0.38-1.60) |  |  |  |  |  |  |  |  |
| > 26.6 (27.8) | 7003 | 15 | 0.69 (0.32-1.49) |  |  |  |  |  |  |  |  |
| P-trend |  |  | 0.563 |  |  |  |  |  |  |  |  |
| per 5 kg/m^2^ | 31022 | 64 | 0.91 (0.58-1.43) |  |  |  |  |  |  |  |  |
| **Women – colon** | |  |  |  |  |  |  |  |  |  |  |
| <22.8 (21.5) | 9014 | 50 | 1.00 (ref.) | 29 | 1.00 (ref.) | 53 | 1.00 (ref.) | 43 | 1.00 (ref.) |  |  |
| 22.8-24.7 (23.8) | 8914 | 46 | 0.86 (0.53-1.40) | 27 | 0.99 (0.54-1.79) | 39 | 0.68 (0.42-1.10) | 33 | 0.76 (0.45-1.30) |  |  |
| 24.8-27.0 (25.7) | 8141 | 64 | 1.35 (0.78-2.32) | 30 | 1.24 (0.62-2.45) | 37 | 0.66 (0.36-1.18) | 23 | 0.59 (0.30-1.14) |  |  |
| >27.0 (29.2) | 8158 | 57 | 1.17 (0.57-2.41) | 26 | 1.16 (0.50-2.72) | 41 | 0.69 (0.32-1.48) | 30 | 0.83 (0.36-1.88) |  |  |
| P-trend |  |  | 0.351 |  | 0.588 |  | 0.330 |  | 0.437 |  |  |
| per 5 kg/m^2^ | 34228 | 217 | 1.43 (1.04-1.96) | 112 | 1.22 (0.79-1.89) | 170 | 0.93 (0.65-1.35) | 129 | 0.99 (0.64-1.53) |  |  |
| **Women – rectum** | |  |  |  |  |  |  |  |  |  |  |
| <22.8 (21.5) | 9014 | 18 | 1.00 (ref.) |  |  |  |  |  |  |  |  |
| 22.8-24.7 (23.8) | 8914 | 7 | 0.51 (0.20-1.29) |  |  |  |  |  |  |  |  |
| 24.8-27.0 (25.7) | 8141 | 9 | 0.85 (0.32-2.30) |  |  |  |  |  |  |  |  |
| >27.0 (29.2) | 8158 | 20 | 2.81 (0.87-9.07) |  |  |  |  |  |  |  |  |
| P-trend |  |  | 0.131 |  |  |  |  |  |  |  |  |
| per 5 kg/m^2^ | 34228 | 54 | 1.63 (0.95-2.77) |  |  |  |  |  |  |  |  |
|  |  |  |  |  |  |  |  |  |  |  |  |
| **Clothing size: range (median)** | | | |  |  |  |  |  |  |  |  |
|  |  |  |  |  |  |  |  |  |  |  |  |
| **Men – colon** | |  |  |  |  |  |  |  |  |  |  |
| ≤50 (50) | 10903 | 73 | 1.00 (ref.) | 40 | 1.00 (ref.) | 30 | 1.00 (ref.) | 18 | 1.00 (ref.) |  |  |
| 52 (52) | 9750 | 84 | 1.03 (0.71-1.48) | 52 | 1.21 (0.76-1.91) | 29 | 0.78 (0.42-1.42) | 23 | 1.18 (0.56-2.46) |  |  |
| 54 (54) | 5156 | 48 | 0.96 (0.61-1.53) | 22 | 0.89 (0.48-1.63) | 21 | 0.87 (0.42-1.77) | 12 | 1.06 (0.43-2.60) |  |  |
| ≥56 (56) | 2619 | 29 | 0.90 (0.48-1.70) | 17 | 1.08 (0.51-2.28) | 9 | 0.54 (0.20-1.46) | 7 | 1.03 (0.31-3.42) |  |  |
| P-trend |  |  | 0.739 |  | 0.848 |  | 0.364 |  | 0.976 |  |  |
| per 2 sizes | 28428 | 234 | 0.99 (0.77-1.26) | 131 | 1.09 (0.79-1.51) | 89 | 0.90 (0.59-1.36) | 60 | 1.22 (0.72-2.06) |  |  |
| **Men – rectum** | |  |  |  |  |  |  |  |  |  |  |
| ≤50 (50) | 10903 | 21 | 1.00 (ref.) |  |  |  |  |  |  |  |  |
| 52 (52) | 9750 | 17 | 0.91 (0.46-1.79) |  |  |  |  |  |  |  |  |
| 54 (54) | 5156 | 17 | 1.76 (0.80-3.91) |  |  |  |  |  |  |  |  |
| ≥56 (56) | 2619 | 9 | 1.83 (0.71-4.75) |  |  |  |  |  |  |  |  |
| P-trend |  |  | 0.105 |  |  |  |  |  |  |  |  |
| per 2 sizes | 28428 | 64 | 1.01 (0.70-1.47) |  |  |  |  |  |  |  |  |
| **Women – colon** | |  |  |  |  |  |  |  |  |  |  |
| ≤40 (40) | 6574 | 35 | 1.00 (ref.) | 24 | 1.00 (ref.) | 29 | 1.00 (ref.) | 32 | 1.00 (ref.) |  |  |
| 42 (42) | 8582 | 54 | 0.89 (0.55-1.44) | 25 | 0.62 (0.33-1.18) | 47 | 1.27 (0.73-2.21) | 35 | 0.73 (0.40-1.32) |  |  |
| 44 (44) | 9270 | 58 | 0.73 (0.43-1.24) | 33 | 0.62 (0.31-1.23) | 45 | 1.06 (0.55-2.05) | 25 | 0.42 (0.20-0.89) |  |  |
| ≥46 (46) | 9454 | 70 | 0.65 (0.33-1.28) | 30 | 0.44 (0.19-1.06) | 49 | 1.24 (0.55-2.79) | 37 | 0.57 (0.23-1.42) |  |  |
| P-trend |  |  | 0.165 |  | 0.111 |  | 0.838 |  | 0.109 |  |  |
| per 2 sizes | 33880 | 217 | 1.07 (0.76-1.49) | 112 | 1.12 (0.72-1.75) | 170 | 1.15 (0.78-1.68) | 129 | 0.85 (0.54-1.34) |  |  |
| **Women – rectum** | |  |  |  |  |  |  |  |  |  |  |
| ≤40 (40) | 6574 | 10 | 1.00 (ref.) |  |  |  |  |  |  |  |  |
| 42 (42) | 8582 | 9 | 0.55 (0.21-1.43) |  |  |  |  |  |  |  |  |
| 44 (44) | 9270 | 12 | 0.61 (0.22-1.63) |  |  |  |  |  |  |  |  |
| ≥46 (46) | 9454 | 23 | 1.08 (0.35-3.36) |  |  |  |  |  |  |  |  |
| P-trend |  |  | 0.722 |  |  |  |  |  |  |  |  |
| per 2 sizes | 33880 | 54 | 0.66 (0.37-1.17) |  |  |  |  |  |  |  |  |
|  | | | | | | | | | | | |
| Abbreviations: HR, hazard ratio; CI, confidence interval; CRC, colorectal cancer; (d/p)MMR, mismatch repair (deficient/proficient); NLCS, Netherlands Cohort Study; mut, mutated; BMI, body mass index.  ^a^Hazard Ratios were adjusted for age (years; continuous), non-occupational physical activity (minutes/day; continuous), total energy intake (kcal/day; continuous), family history of CRC (yes/no), alcohol consumption (0; 0.1-4; 5-14; >15 g/day), processed meat intake (g/day; continuous), red meat intake (g/day; continuous). Age was included as a time-varying covariate. BMI models were additionally adjusted for height (cm; continuous). Mutual adjustment: clothing-size (size; continuous) was included in BMI models, and BMI (kg/m^2^; continuous) was included in clothing-size models.  ^b^Analyses for subgroups with <50 cases were not performed. | | | | | | | | | | | |
|  | | | | | | | | | | | |
